# Genetic Divergence of Lineage-Specific Tandemly Duplicated Gene Clusters in Four Diploid Potato Genotypes

**DOI:** 10.3389/fpls.2022.875202

**Published:** 2022-05-11

**Authors:** Venkata Suresh Bonthala, Benjamin Stich

**Affiliations:** ^1^Institute of Quantitative Genetics and Genomics of Plants, Heinrich Heine University of Düsseldorf, Düsseldorf, Germany; ^2^Max Planck Institute for Plant Breeding Research, Köln, Germany; ^3^Cluster of Excellence on Plant Sciences, From Complex Traits Towards Synthetic Modules, Düsseldorf, Germany

**Keywords:** tandem duplication, lineage-specific duplicated genes, gene expression, whole-genome duplication, agronomic traits

## Abstract

Potato (*Solanum tuberosum* L.) is the most important non-grain food crop. Tandem duplication significantly contributes to genome evolution. The objectives of this study were to (i) identify tandemly duplicated genes and compare their genomic distributions across potato genotypes, (ii) investigate the bias in functional specificities, (iii) explore the relationships among coding sequence, promoter and expression divergences associated with tandemly duplicated genes, (iv) examine the role of tandem duplication in generating and expanding lineage-specific gene families, (v) investigate the evolutionary forces affecting tandemly duplicated genes, and (vi) assess the similarities and differences with respect to above mentioned aspects between cultivated genotypes and their wild-relative. In this study, we used well-annotated and chromosome-scale *de novo* genome assemblies of multiple potato genotypes. Our results showed that tandemly duplicated genes are abundant and dispersed through the genome. We found that several functional specificities, such as disease resistance, stress-tolerance, and biosynthetic pathways of tandemly duplicated genes were differentially enriched across multiple potato genomes. Our results indicated the existence of a significant correlation among expression, promoter, and protein divergences in tandemly duplicated genes. We found about one fourth of tandemly duplicated gene clusters as lineage-specific among multiple potato genomes, and these tended to localize toward centromeres and revealed distinct selection signatures and expression patterns. Furthermore, our results showed that a majority of duplicated genes were retained through sub-functionalization followed by genetic redundancy, while only a small fraction of duplicated genes was retained though neo-functionalization. The lineage-specific expansion of gene families by tandem duplication coupled with functional bias might have significantly contributed to potato’s genotypic diversity, and, thus, to adaption to environmental stimuli.

## Introduction

Gene duplication is thought to have significantly contributed to the evolution of genetic and morphological diversity, and speciation in eukaryotes (Ohno, 1970). Plant genomes contain a significant proportion of duplicated genes that are formed by various mechanisms, including single-gene duplications and larger chromosomal regions or whole-genome duplication (WGD or polyploidization; Freeling and Thomas, 2006; Flagel and Wendel, 2009). WGD is prevalent in the plant kingdom and involves duplication of all nuclear genes of an organism. This in turn leads to a sudden increase in both genome size and the entire gene set (Moghe and Shiu, 2014; Salman-Minkov et al., 2016). Many angiosperm lineages experienced repeated WGD events throughout their evolutionary history, and genome sequencing continues to report new events in various plant species (Schmutz et al., 2010; Zhang et al., 2011; Li et al., 2015). Recent WGD that have occurred in lineages of crop species, including soybean, wheat, and cotton, have contributed to important traits, such as nodulation and oil production (Schmutz et al., 2010), grain quality (Zhang et al., 2011), and spinnable fibers (Li et al., 2015), respectively.

In addition to WGD, single-gene duplications, such as tandem, proximal, dispersed, DNA-transposed, and retrotransposed duplications are also prevalent in plant genomes. These contribute to the expansion and evolution of multigene families (Cannon et al., 2004; Freeling, 2009; Qiao et al., 2018, 2019). Tandemly duplicated genes (TDG) are present next to the original copy or are intervened by several unrelated genes in the same genomic neighborhoods and often occur as a result of unequal crossing over followed by inversions or transposon activities (Freeling, 2009). Furthermore, these genes are found to be scattered throughout the genome but the majority tend to localize toward terminal regions of the chromosomes (Jiang et al., 2013; Kono et al., 2018; Qiao et al., 2018). TDGs exhibit bias in functional specificities to generate functional novelties in the genome (Jiang et al., 2013; Qiao et al., 2018). In addition, tandem duplication generates lineage-specific gene duplicates followed by their expansion among evolutionarily closed (Kono et al., 2018) and distant species (Hanada et al., 2008) for adaptive response to environmental stimuli. However, it is unclear whether tandem duplication creates bias in functional specificities and lineage-specific gene duplicates between cultivated species and their wild relatives.

Tandemly duplicated genes may experience different evolutionary fates such as (i) loss of one of duplicated gene copy *via* pseudogenization and/or accumulation of deleterious mutations (Panchy et al., 2016), (ii) retention of both duplicated genes due to selection for genetic redundancy that may be beneficial (Panchy et al., 2016), (iii) retention of duplicated genes simply because there has been insufficient time for one copy to be removed/mutated or due to genetic drift (Panchy et al., 2016), and (iv) retention of both duplicated genes because of a selective advantage either due to the existing or the novel functions (Panchy et al., 2016). The retention of both duplicated genes because of selective advantage due to the existing functions can be explained by gene dosage (Ohno, 1970), sub-functionalization (Force et al., 1999), dosage balance (Freeling and Thomas, 2006), and paralog interference (Baker et al., 2013) models. Similarly, both duplicated genes can be retained because of selective advantage due to novel functions and can be explained by neo-functionalization (Ohno, 1970) and escape from adaptive conflict (Des Marais and Rausher, 2008) models. Among the above-mentioned models, both sub- and neo-functionalization provide testable hypotheses suggesting that the sub-functionalized gene copies show divergence in expression across tissues and are expected to undergo purifying selection (i.e., *K*_a_/*K*_s_ < 1) because the functions of ancestral gene have become divided among the daughter copies (Force et al., 1999; Duarte et al., 2006; Cusack and Wolfe, 2007; Ma et al., 2015), whereas neo-functionalized gene copies undergo positive selection (i.e., *K*_a_/*K_s_* > 1) because gain of a novel function by one of gene copy that contributes to better fitness (Ohno, 1970; Blanc and Wolfe, 2004). Based on these testable hypotheses, Roulin et al., 2013 unraveled the contribution of sub- and neo-functionalization in retention of duplicated genes in soybean, and found that 50% of paralogs have undergone expression sub-functionalization, while a small fraction of paralogs has been neo-functionalized. However, it is unclear whether sub- or/and neo-functionalization play a role in retention of TDGs between cultivated species and their wild relatives. In addition, it is also unclear whether tandem duplication creates different proportion of duplicated genes between cultivated species and their wild relatives.

Potato (*Solanum tuberosum*. L) is a highly heterozygous autotetraploid species, and is the world’s most important non-grain food crop with a worldwide production of 370 million metric tons *per annum* (FAO, 2019). Wang et al. (2018) focused on comparative analysis of DNA methylation patterns between duplicated genes of potato and tomato, and found DNA methylation divergence between duplicated genes. Recently, Qiao et al. (2019) investigated the signatures of selection, expression divergence, and gene conversion underlying evolution of duplicated genes across 141 plant species including potato. However, these two studies did not address various aspects associated with TDGs in potato. This includes the genomic distribution, bias in functional specificities, the influence of evolutionary forces, and relationships among coding sequence, promoter and expression divergences associated with TDGs. Further, the role of tandem duplication in generating lineage-specific gene families and their expansions, as well as the factors contributing to the retention of TDGs, were not studied in potato yet.

The objectives of our study were to (i) identify TDGs and compare their genomic distributions across potato genotypes, (ii) investigate the bias in functional specificities, (iii) explore the relationships among coding sequence, promoter, and expression divergences of TDGs, (iv) examine the role of tandem duplication in generating and expanding lineage-specific gene families, (v) investigate the evolutionary forces affecting the TDGs, and (vi) assess the similarities and differences with respect to above mentioned aspects between cultivated genotypes and their wild-relative.

## Materials and Methods

### Data Sources

Thousands of potato cultivars exists and most of them are tetraploid (2n = 4x = 48; FAO, 2008). However, chromosome level genome assemblies for tetraploid potato clones became only recently available (Hoopes et al., 2022), after the analyses for this study were finalized. Instead, we used four tuber-bearing diploid potato clones belonging to cultivated, non-cultivated, and wild potato species for which chromosome-level genome assemblies are available. The cultivated potato *Solanum tuberosum* ssp. *tuberosum* L. is represented in our study by a diploid clone (hereafter referred to as dAg) which was derived from the tetraploid elite potato cultivar Agria (tAg; Freire et al., 2021). The non-cultivated potato clones include *Solanum tuberosum* L. DM1-3516 R44 (hereafter referred to as DM), which is a doubled monoploid clone derived from the group Phureja (Pham et al., 2020) and *Solanum tuberosum* L. RH89-039-16 (hereafter referred to as RH) which is a diploid clone derived from a cross between a dihaploid and a diploid potato (Zhou et al., 2020). The wild clone in our study is *Solanum chacoense* M6 (hereafter referred to as M6; Leisner et al., 2018). Both the sequence (genome and gene) and annotation files for DM (version 6.1), RH, and M6 were downloaded from http://potato.plantbiology.msu.edu and for dAg was obtained from Freire et al. (2021). In addition, we obtained transposable elements (TEs) annotation for dAg and DM from the above-mentioned sources. Due to the lack or absence of chromosome-level TE annotation for M6 and RH, we excluded TE annotation for these two genotypes from the analyses.

### Improving the Gene Annotation for dAg

In this study, we improved the existing gene annotation for dAg using the PASA pipeline v2.5.0 (Haas et al., 2003) followed by classifying the resulting annotation into full-length and partial gene models using AGAT v0.8.0 (Dainat et al., 2022). In that procedure, we used transcriptome datasets generated as part of dAg genome sequencing (Freire et al., 2021) to improve the existing gene annotations.

### Functional Annotation, Orthology Prediction, and Filtering Transposons

For reasons of consistency, we have performed functional annotation for the longest isoform of high-confidence genes of all four potato genomes using the AHRD pipeline.^1^ Orthologs among the four potato genomes were predicted by feeding protein sequences of the longest isoform of high-confidence genes to OrthoFinder v2.5.4 (Emms and Kelly, 2019). As, high-confidence genes, we considered those genes for which expression/functional evidence was available and that had full-length without internal stop-codons. Hence, annotated partial/pseudogenes were ignored in our study. Furthermore, we annotated transposon (TE) or TE-related genes in all four potato genomes using the approach described by Jayakodi et al. (2020). Briefly, this approach involves two stages to annotate TEs in all annotated genes. The first stage involves searching for keywords and PFAM IDs related to TEs in the functional annotation obtained from AHRD and classify each gene as either TE or non-TE gene. The second stage involves combining the orthogroup (OG) information for each gene obtained from OrthoFinder with the curated genes of the first stage. In the last step, we evaluated whether each OG is classified as non-TE OG based on the criteria that the OG contains less than 30% of TE genes and the mean AHRD score of OG is ≥2, otherwise the OG is classified as TE OG.

### Identification of TDG Clusters

Tandemly duplicated genes clusters were identified among the non-TE genes of each potato genome separately using the methodology described by Jayakodi et al. (2020). In our study, we restricted our analyses to non-TE genes with a known chromosomal location. Briefly, this approach involves the identification of homologous gene pairs present on the same chromosome using all vs. all BlastN (Altschul et al., 1990) of coding sequences (CDS) of the longest iso-form of high-confidence gene models. This is followed by finding TDG clusters based on the following thresholds: *e*-value cut off of 1e^−10^, bit score ratio of ≥30%, and coverage of both query and subjects of ≥50%. In this study, we defined TDGs as the homologous genes present on the same chromosome which are intervened by up to 10 genes. TDGs (or TDG pair) correspond to single genes (or gene pairs) that belong to a TDG cluster. A TDG cluster corresponds to a group of TDG pairs.

Further, the variation in density of TDGs across the respective genomes were explained by fitting a general linear model against the density of various genomic features such as all non-TE genes, DNA transposable elements (TEs), and RNA TEs using R v3.6.1.^2^ In the next step, the residuals of these models were tested against a uniform distribution using a Kolmogorov–Smirnov (KS) test in order to evaluate whether the TDGs were distributed uniformly across the genome after adjusting for the distribution effects of the above-mentioned genomic features. In addition, Pearson’s correlation coefficients between density of TDGs and the above-mentioned genomic features were computed. All density calculations were performed in 1 Mb windows across the genome. For TDG also, 1.5 Mb windows were considered. Density was defined as the proportion of bases in each window that corresponded to the respective genomic feature. TDG clusters were categorized based on their level of sharing across four potato genomes into core, i.e., present in all four potato genomes, shared, i.e., present in more than one potato genome but absent in at least one potato genome, and private (or lineage-specific) clusters, i.e., present in a single potato genome.

### Enrichment of Pfam Domains and Gene Ontology Terms Among TDGs

Both Pfam domain and Gene Ontology (GO) term information was extracted from the output of AHRD for each potato genome. For each detected Pfam domain, we calculated the number of proteins present among the proteins of TDGs followed by performing the protein domain enrichment analysis using Fisher exact test (Fisher, 1992). FDR corrected values of *p* < 0.05 were considered as significant. Enrichment of GO terms was performed using GOATOOLS (Klopfenstein et al., 2018).

### Gene Expression Quantification and Estimating Expression Divergence

For this analysis, publicly available RNA-Seq datasets from NCBI SRA^3^ were used (Supplementary Tables S5–S8). The raw-reads were filtered for low-quality and trimmed adapter sequences using Trimmomatic v0.39 (Bolger et al., 2014) with the following parameters: (1) adapters were removed using for pair-end: ILLUMINACLIP:TruSeq3-PE.fa:2:30:10:8:true, for single-end: ILLUMINACLIP:TruSeq3-SE.fa:2:30:10:8; (2) removing leading and trailing low-quality or *N* bases using LEADING:3 TRAILING:3; (3) scanning the read with a four-base wide sliding window, cutting when the average quality per base drops below 20 (SLIDINGWINDOW:4:20); and (4) selecting reads with at least 50 (for pair-end: MINLEN:50) or 36 bases long (for single-end: MINLEN:36). Only, RNA-Seq datasets with at least 50% of high-quality reads after trimming were chosen for further analysis. The high-quality reads were used to estimate transcripts abundances on a gene level (i.e., averaging across the alleles present at the respective gene) using Kalisto v0.46.1 (Bray et al., 2016) with default parameters (for single-ends: -l 200 -s 20) and obtained transcript per million (TPM) values. For all examined four genomes, we classified a gene as expressed, if TPM was >0.5 in at least one RNA-Seq dataset, otherwise classified as unexpressed. In the next step, we evaluated expression breadth, i.e., the number of RNA-Seq datasets in which the gene was expressed. Further, the expressed genes were used to calculate the expression level and expression specificity. The expression level was defined as the mean value of TPM across RNA-Seq datasets for each gene. The expression specificity was measured as described by Yang and Gaut (2011) and ranged from 0 to 1, with a higher value indicating higher specificity, i.e., higher variation in expression across RNA-Seq datasets. If a gene is expressed in a single library only, the expression specificity is 1. In contrast, if a gene is expressed equally in all RNA-Seq datasets, the expression specificity is 0.

### Collection of Promoter Sequences and Estimating Promoter Divergence

For each potato genome and each gene, we considered the non-overlapping 1 Kb sequence upstream of the transcription start site (TSS) as putative promoter sequence and retrieved it from the respective potato genomes using BEDTools v2.27.1 (Quinlan and Hall, 2010). The promoter sequences for each tandem duplicate gene pair were aligned using the matcher program of EMBOSS v6.6.0.0 (Rice et al., 2000) to compute promoter similarity (Ps). We excluded promoter sequences that contained unknown nucleotides (N).

### Computing *K*_a_, *K*_s_, and *K*_a_/*K*_s_ Values

For each TDG pair, the amino acid sequences were aligned using MAFFT v7.453 (Katoh and Standley, 2013) followed by reverse translation into nucleotide alignment using PAL2NAL v14 (Suyama et al., 2006). Finally, the nucleotide alignments were used to compute *K*_a_, *K*_s_, and *K*_a_/*K*_s_ values using the Gamma-MYN method of *K*_a_*K*_s__Calculator v2.0 (Wang et al., 2010). As the high number of reversions or multiple substitutions at synonymous sites reduces accuracy and reliability for rate estimation (Vanneste et al., 2013), we excluded *K*_s_ values >2 from the analysis.

### Correlation Analyses Among Promoter, Protein, and Expression Divergences

Pearson’s correlation coefficient (*r*) was calculated across all TDG pairs between (i) expression and promoter divergence, (ii) expression divergence and age of duplicate pairs, (iii) expression divergence and *K*_a_/*K*_s_ ratios, and (iv) promoter divergence and age of duplicate pairs using SciPy library (Virtanen et al., 2020) in Python v3.8.5.^4^

## Results

### Annotation of Transposon-Related Genes in Potato Genomes

The recently published gene annotation of the diploid clone dAg derived from the elite variety Agria does not contain information about isoforms and partial gene models. Hence, we first improved the existing gene annotation of dAg using the PASA pipeline (Haas et al., 2003) by utilizing the available Iso-Seq data of Agria (tAg; Freire et al., 2021) and obtained 44,464 gene models with 58,734 isoforms. We then filtered out partial gene models using AGAT tools (Dainat et al., 2022) and obtained 39,088 full-length gene models with 53,352 isoforms (referred as full-length set in Table 1A). This new annotation contains a significantly higher number of gene models than the high-confidence annotation of DM v6.1 (Pham et al., 2020), and a slightly higher number of gene models than the annotation of M6 (Leisner et al., 2018) and RH (Zhou et al., 2020; Table 1B).

**Table 1:** **(A)** Improved gene annotation of dAg; **(B)** Non-TE genes of potato genomes.

Genomic feature	Old annotation	Working set	Full-length set	Representative set	Partial set
Table 1A					
# Genes	44,952	44,464	39,088	39,088	5,382
# mRNAs	44,952	58,734	53,352	39,088	5,382
# CDSs	220,904	354,174	332,602	195,622	21,572
# Exons	226,161	374,932	353,161	201,589	21,771
# Introns	NA	316,198	299,809	162,501	16,389
# 5`-UTRs	17,300	44,596	43,643	20,385	953
# 3`-UTRs	16,118	39,268	39,049	19,432	219
Table 1B					
Potato Genotype		# Genes before TE Filtering	# Genes after TE filtering	% Genes TEs	
dAg		39,088	33,934	13.19	
DM		32,917	31,494	4.32	
M6		37,740	35,330	6.39	
RH		37,115	31,249	15.8	
Total		146,860	132,007	10.11	

Genes left after TE filtering were used for all down-stream analyses. The working set, Full-length set, Representative set, and Partial sets indicate all annotated gene models, gene models with full length genes, gene models with longest isoforms, and gene models with partial genes, respectively.

To ensure that the results of our analyses can be compared across all four potato genomes, the functional annotation for all four potato genomes was performed using the AHRD pipeline.^5^ This pipeline assigns a quality score in the form of a three-character string, where each character is either “*” if respective criteria is met or “-” otherwise, for each annotated gene to indicate how confident the assigned annotation is. The “*” in first position indicates bit score, and *e*-value of the blast result are >50 and 1e^−10^, respectively. The “*” in second position indicates overlap of the blast result is >60%, and the “*” in third position indicates top token score of assigned Human-Readable-Description is >0.5. In our study, we selected annotation with a quality score of at least two stars as best annotation, i.e., at least two out of three criteria should meet by the annotated gene. Overall, AHRD assigned functions to 84.31% of the genes of all four potato genomes with at least two stars, whereas, AHRD was unable to annotate 13.13% of the genes of all four potato genomes (Supplementary Table S1). We also estimated orthologs and orthogroups among the four potato genomes using OrthoFinder (Emms and Kelly, 2019) and obtained 28,647 orthogroups representing 93.5% of the genes of all potato genomes. A total of 16,107 orthogroups contained genes from all four potato genomes, of which 9,563 were single-copy orthogroups (Supplementary Table S2). Finally, we annotated 10.11% of the genes (Table 1B) of all four potato genomes as TE or TE-related genes using the approach of Jayakodi et al. (2020).

### Systematic Identification of TDG Clusters in Four Potato Genomes

**Figure 1:**
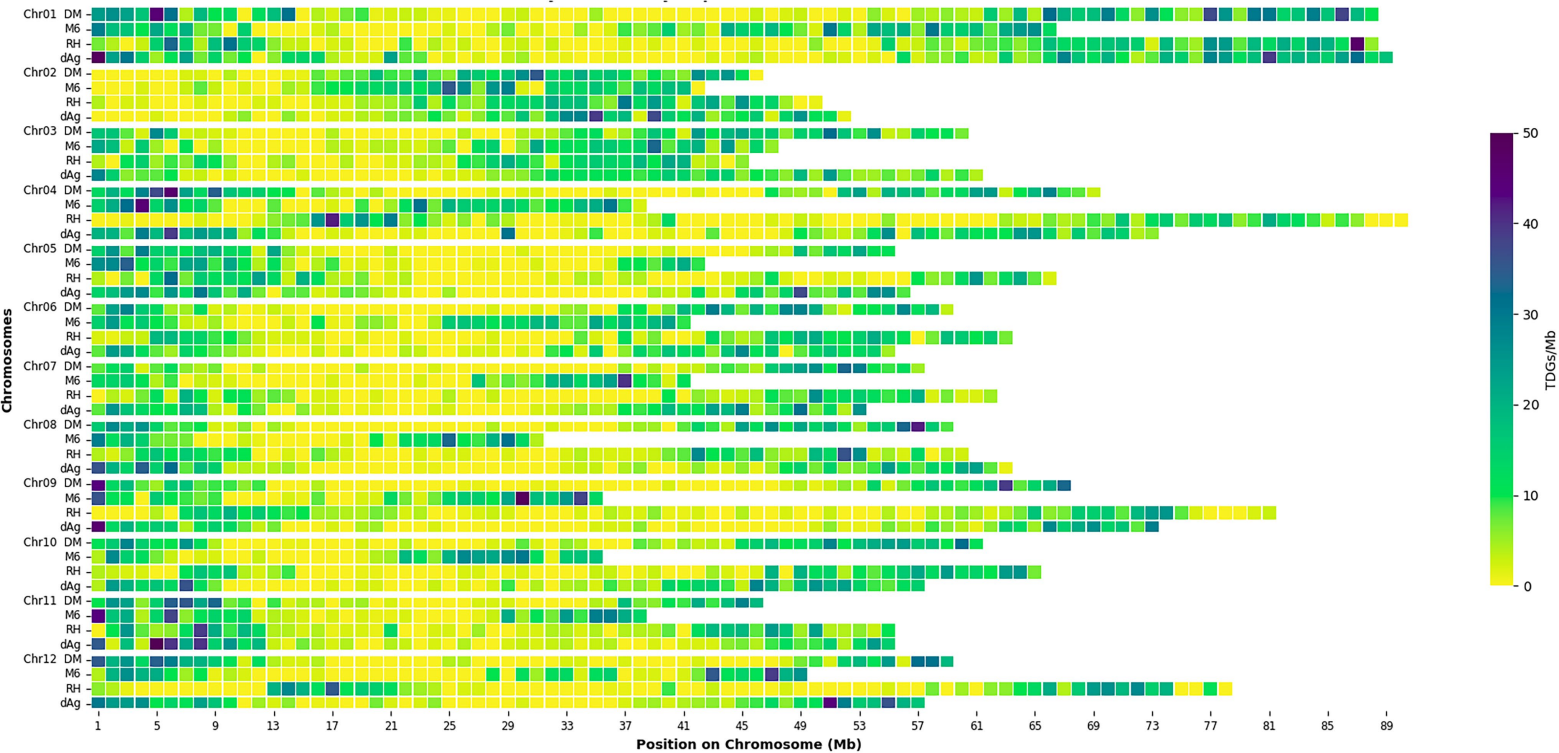
Distribution of density of tandemly duplicated genes per 1 Mb across each potato genome. Square boxes between rows do not correspond to sequence alignment.

**Figure 2:**
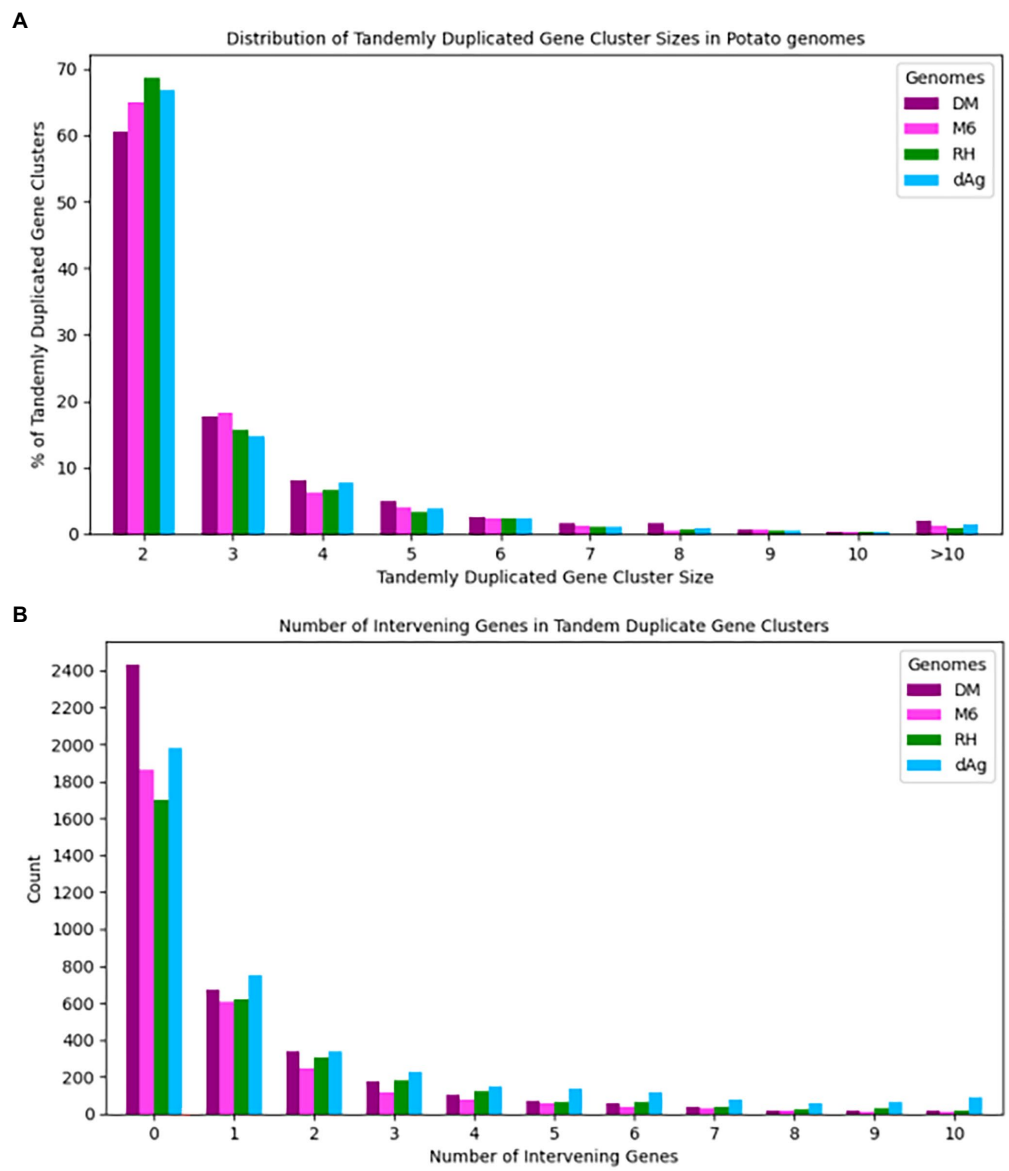
**(A)** Distribution of cluster sizes vs. percent of clusters across four potato genomes and **(B)** Number of intervening genes vs. number of tandem duplicated gene pairs across four potato genomes.

Tandemly duplicated genes clusters were identified among the non-TE genes of each potato genome. In total, 2,090, 1,867, 1,661, and 1,832 TDG clusters were identified in dAg, DM, M6, and RH, respectively. The percentage of annotated genes in TDG clusters of the total number of genes were 18.67, 18.52, 16.83, and 16.06% in dAg, DM, M6, and RH, respectively (Table 2). The availability of multiple high-quality *de novo* potato genome assemblies allowed us to determine the consistency of various characteristics of TDG clusters across potato genomes. The identified TDGs were dispersed throughout the genome and shown to have a similar distribution across the four potato genomes (Figure 1). Moreover, the density distribution of TDGs with both 1 and 1.5 Mb sliding-windows resulted in same density distribution pattern across the four potato genomes (Figure 1; Supplementary Figure S5). Overall, the density of TDGs across the genome was significantly (value of *p* < 2.2e^−16^) associated with the density of non-TE genes across the respective potato genomes. The correlation coefficients were 0.6, 0.68, 0.61, and 0.63 for dAg, DM, M6, and RH, respectively. The same observation was made for the densities of both DNA and RNA TEs. After correcting for these densities using a general linear model, the density of TDG showed a significant (value of *p* < 2.2e^−16^) deviation from a uniform distribution in each potato genome. The KS test statistics (*D*) are 0.38, 0.38, 0.41, and 0.57 for dAg, DM, M6, and RH, respectively. Chromosome 1 of all potato genomes harbored the highest number of TDG clusters, while Chromosomes 6, 5, 8, and 7 harbored the lowest number of TDG clusters in dAg, DM, M6, and RH, respectively (Supplementary Table S3). A similar distribution in terms of the size of TDG clusters was observed across the four potato genomes, and the majority of the TDG clusters comprised two genes (Figure 2A). Further, the majority of TDG pairs within TDG clusters did not contain intervening genes, and moreover, DM contained the highest number of TDG pairs without intervening genes among the four potato genomes (Figure 2B). For dAg, a higher proportion of TDGs with two exons was observed compared to the other three genomes. For the latter, the proportion of TDG with single exons was higher compared to that of non-tandemly duplicated genes (Supplementary Figure S1).

**Table 2:** Summary of identified putative tandemly duplicated gene clusters in potato genomes.

Description	dAg	DM	M6	RH
Number of non-TE genes	33,934	31,494	35,330	31,249
Number of non-TE genes (Known Location)	32,555	31,410	28,210	31,249
Number of TDG Clusters	2090	1867	1,661	1832
Number of genes in TDG Clusters	6,078	5,817	4,748	5,018
Percentage of genes in TDG clusters	18.67	18.52	16.83	16.06
Number of Orthogroups	2,654	2,576	2,218	2,352
Percent of TDG clusters with two genes	66.89	60.47	64.90	68.72
Largest TDG cluster	36	31	23	21
Percent of TDGs with Pfam domains	83.42	91.47	86.73	78.68
Number of Pfam Protein Domains identified in TDGs	917	805	706	756
Number of Pfam Protein Domains Enriched in TDGs	46	69	60	61
Number of Pfam Domains Enriched in TDGs with *K_a_*/*K_s_* > 1	30	46	30	24
Number of GO terms identified in TDGs	1901	2097	1783	1,634
Significantly Enriched BP terms in TDGs	190	230	199	154
Significantly Enriched BP terms in TDGs with *K*_a_/*K*_s_ > 1	108	162	127	74
Significantly Enriched MF terms in TDGs	135	165	159	102
Significantly Enriched CC terms in TDGs	15	15	14	15
Percent of TDGs expressed (TPM > 0.5)	68.82	81.14	76.98	74.35

### Evolutionary Forces Affecting TDGs

**Figure 3:**
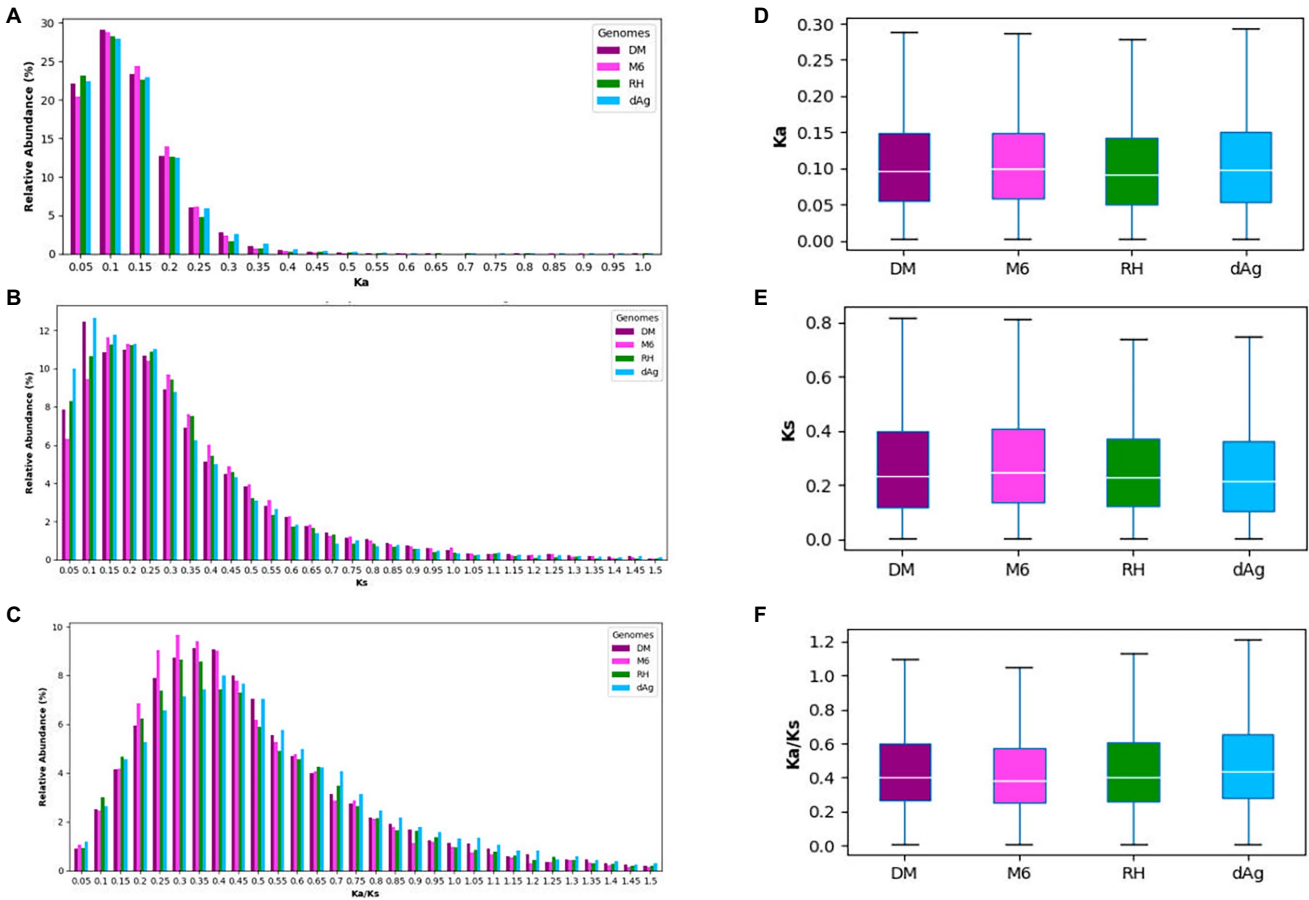
**(A–C)** Distribution of *K*_a_, *K*_s_, and *K*_a_/*K*_s_ for tandemly duplicated genes in DM, M6, RH, and dAg, and sorted into bins of width = 0.1. **(D–F)** Evolutionary patterns of tandemly duplicated genes in DM, M6, RH, and dAg.

Tandemly duplicated genes account for about 18% of the total non-TE genes. Thus, it would be interesting to gain insights into the evolutionary forces that affect the TDGs. Therefore, we examined the sequence divergence in TDGs of each potato genome by estimating *K*_a_ (number of substitutions per nonsynonymous site), *K*_s_ (number of substitutions per synonymous site), and *K*_a_/*K*_s_ ratios, and compared their distributions across the four potato genomes. We observed pronounced peaks at 0.1, between 0.1 and 0.15, as well as between 0.3 and 0.4 for *K*_a_, *K*_s_, and *K*_a_/*K*_s_ distributions, respectively, for all potato genomes (Figures 3A–C). Further, all four potato genomes showed a higher *K*_a_/*K*_s_ values compared to *K*_a_ values (Figures 3D,F). An average of 92.97% TDG pairs showed *K*_a_/*K*_s_ < 1.0 (i.e., negative or purifying selection), while an average of 6.47% showed a *K*_a_/*K*_s_ value >1.0 (i.e., positive selection). In addition, we observed that the cultivated potato genotype dAg contained the highest number of TDG pairs (916) that were under positive selection (i.e., *K*_a_/*K*_s_ > 1), while the wild potato genotype M6 contained the least number (345; Supplementary Table S4).

**Figure 4:**
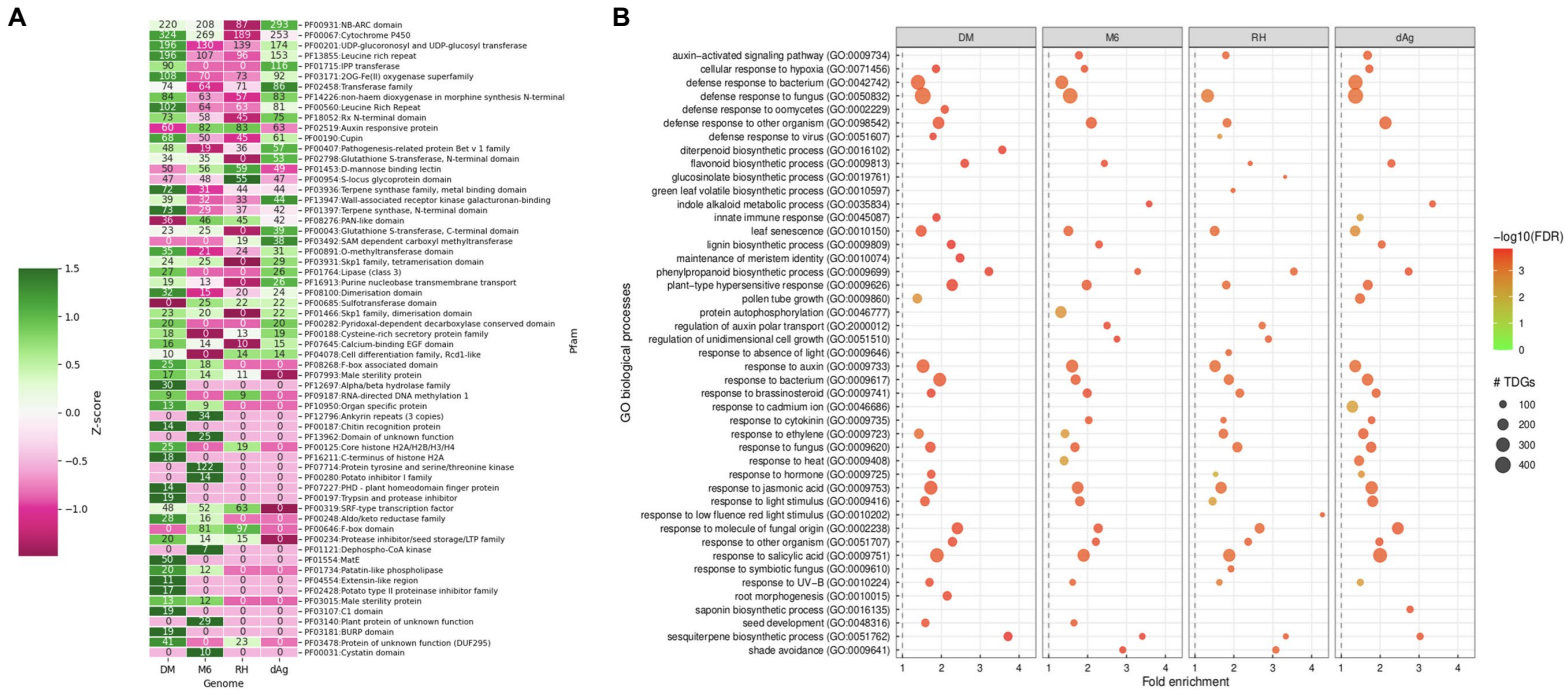
**(A)** Enriched Pfam protein domains in tandemly duplicated genes which are under positive selection (i.e., *K*_a_/*K*_s_ > 1) in DM, M6, RH, and dAg. **(B)** Top 30 GO biological processes enriched in tandemly duplicated genes which are under positive selection (*K*_a_/*K*_s_ > 1) in DM, M6, RH, and dAg.

Furthermore, we investigated the functional specificities of the TDGs by an enrichment analysis to answer whether evolutionary forces drive TDGs in potato toward a specific biological function. First, we used the Pfam protein domain information for an enrichment analysis (DEA). A total of 83.42, 91.47, 86.73, and 78.68% of the identified TDGs contain Pfam domains in dAg, DM, M6, and RH, respectively. Further, a total of 917, 805, 706, and 756 unique Pfam domains were identified in TDGs of dAg, DM, M6, and RH, respectively. Across the four potato genomes, TDGs showed a similar distribution of the number of protein domains they harbor. The majority of TDGs contained a single Pfam domain only (Supplementary Figure S2). The DEA identified that 46, 69, 60, and 61 protein domains in TDGs of dAg, DM, M6, and RH, respectively, were significantly (FDR < 0.05 and a minimum number of 10 TDGs/protein domain) over-represented. The most important protein domains that were enriched included NB-ARC, leucine-rich repeat, pathogenesis-related proteins, UDP-glucosyl transferase, glutathione S-transferase, and auxin-responsive protein IPP transferase. Interestingly, all the enriched protein domains were also differentially enriched (*z*-score) across the four potato genomes (Table 2 and Supplementary Figure S3). Further, a total of 65.21, 66.66, 50, and 39.34% of enriched protein domains were present in positively selected (*K*_a_/*K*_s_ > 1) TDGs of dAg, DM, M6, and RH, respectively (Figure 4A). As alternative approach to identify functional specificities of the TDGs, we performed a GO-term enrichment analysis (GOEA) to identify over-represented gene ontology (GO) terms in TDGs. GOEA identified 190, 230, 199, and 154 statistically significant (FDR < 0.01) biological processes (BP) in dAg, DM, M6, and RH, respectively (Table 2). Further, a total of 56.84, 70.43, 63.81, and 48.05% of enriched BP were present in positively selected (*K*_a_/*K*_s_ > 1) TDGs of dAg, DM, M6, and RH, respectively. The majority of the top 30 BP were associated with defense responses against various biotic conditions (bacteria, fungus, and virus), and stress responses against various abiotic (hypoxia, cadmium, heat, light, and UV-B) stress conditions (Figure 4B). In line with the domain enrichment, the enriched BP were also differentially enriched (fold enrichment) across four potato genomes (Figure 4B).

### Expression Divergence Between TDGs

**Figure 5:**
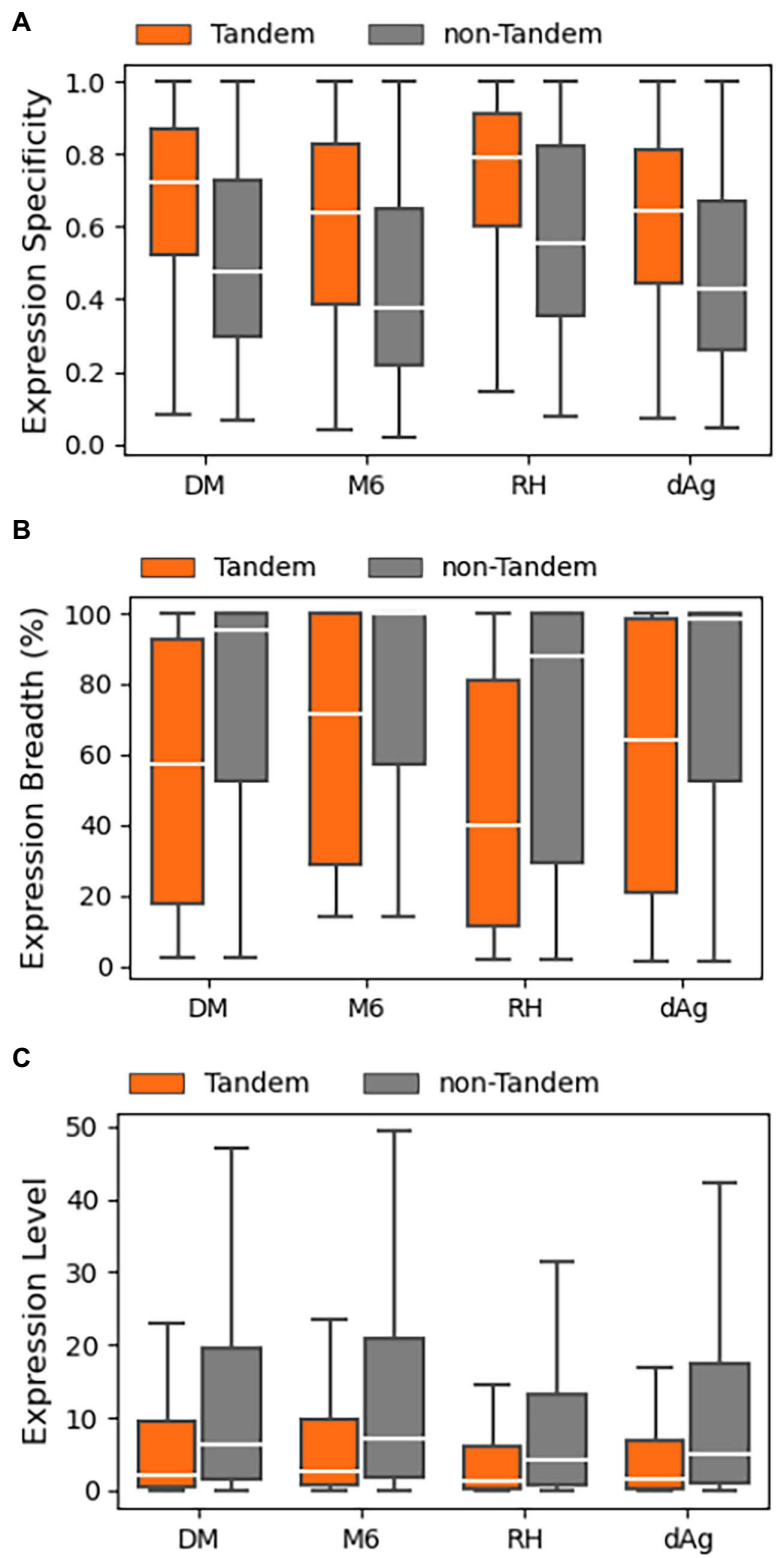
Expression patterns of tandemly duplicated genes in DM, M6, RH, and dAg. **(A)** Expression Specificity, **(B)** Expression Breadth (%), and **(C)** Expression Level.

In this study, we examined patterns of expression divergence between TDGs in four potato genomes using publicly available RNA-Seq datasets of the respective potato genomes except for dAg. In the public domain, only one RNA-Seq dataset was available for tAg, the tetraploid ancestor of dAg, but the dataset did not pass our selection criteria after trimming out low-quality reads to include in the analysis. Consequently, the RNA-Seq datasets generated under various stress conditions (drought, salt, heat, and cold) belonging to different potato cultivars were used to estimate expression of dAg (Supplementary Tables S5–S8). We used log10 transformed transcripts per million (TPM) values obtained from Kallisto (Bray et al., 2016) as a proxy for expression levels. In the next step, we classified each TDG as expressed if TPM > 0.5 in at least one RNA-Seq dataset, otherwise classified as unexpressed. Based on this criterion, we found that 68.82, 81.14, 76.98, and 74.35% of TDGs were expressed in dAg, DM, M6, and RH, respectively (Table 2). Across all potato genomes, TDGs showed higher expression specificities than non-TDGs (Figure 5A), while both expression breadth and levels were lower than non-TDGs (Figures 5B,C).

**Figure 6:**
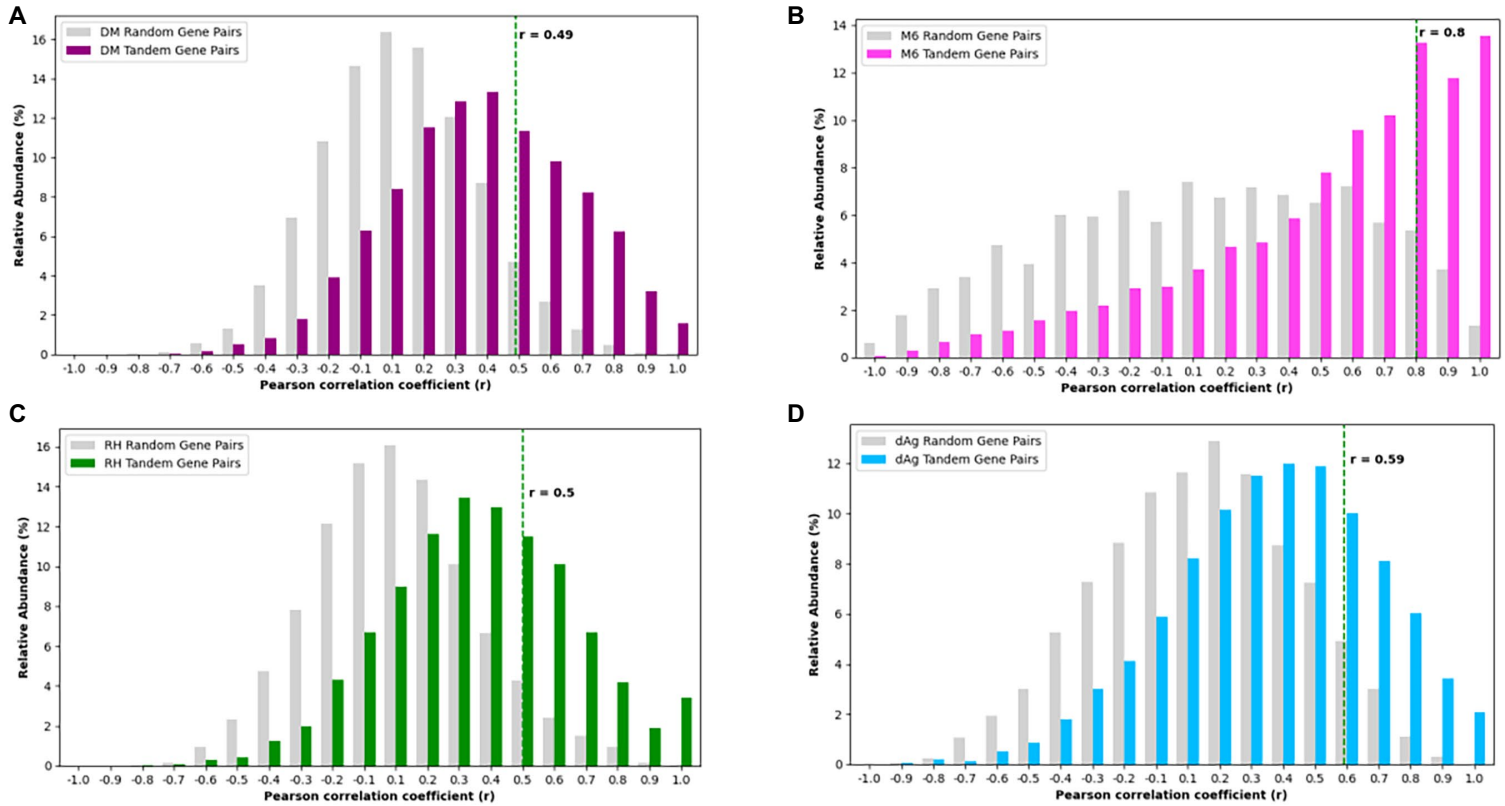
Distributions of Pearson’s correlation coefficient (*r*) between the expression profiles of two copies derived from tandem duplication in **(A)** DM, **(B)** M6, **(C)** RH, and **(D)** dAg. The dashed green line indicates the 95% quantile of *r* distribution of random gene pairs in respective genotypes. *r* < 95% quantile indicates that the gene pairs have diverged expression, while *r* ≥ 95% quantile indicates that the gene pairs have conserved expression in respective potato genomes.

For each potato genome, we classified each TDG pair as expressed TDG pair if both gene copies were expressed, otherwise classified as unexpressed TDG pair. Based on this criterion, we found 46.57, 67.24, 64.29, and 69.08% of TDG pairs were classified as “expressed TDG pairs” in dAg, DM, M6, and RH, respectively (Supplementary Table S9). Further, for each potato genome, we selected expressed TDG pairs and calculated Pearson’s correlation coefficient (r) between expression profiles of both gene copies. As comparison, we calculated r for the same number of randomly selected non-TDG pairs. The TDG pairs of DM, RH, and dAg revealed the same approximate normal distribution of correlation coefficients as the control sample (Figures 6A,C,D), while the TDG pairs of M6 showed for both a distribution of the correlation coefficients that deviated from a normal distribution (Figure 6B). Further, the 95% quantile of the correlation coefficient *r* of randomly selected gene pairs was used as threshold for determining if the two gene copies of a TDG pair have diverged expression, i.e., if *r* > = 95% quantile, then the TDG pair have a similar expression, while *r* < 95% quantile, then the TDG pair have diverged expression. Based on this criterion, 79.56, 69.95, 74.55, and 73.66% of expressed TDG pairs showed diverged expression in dAg, DM, M6, and RH, respectively (Supplementary Table S9).

### Promoter Divergence Between TDGs

**Figure 7:**
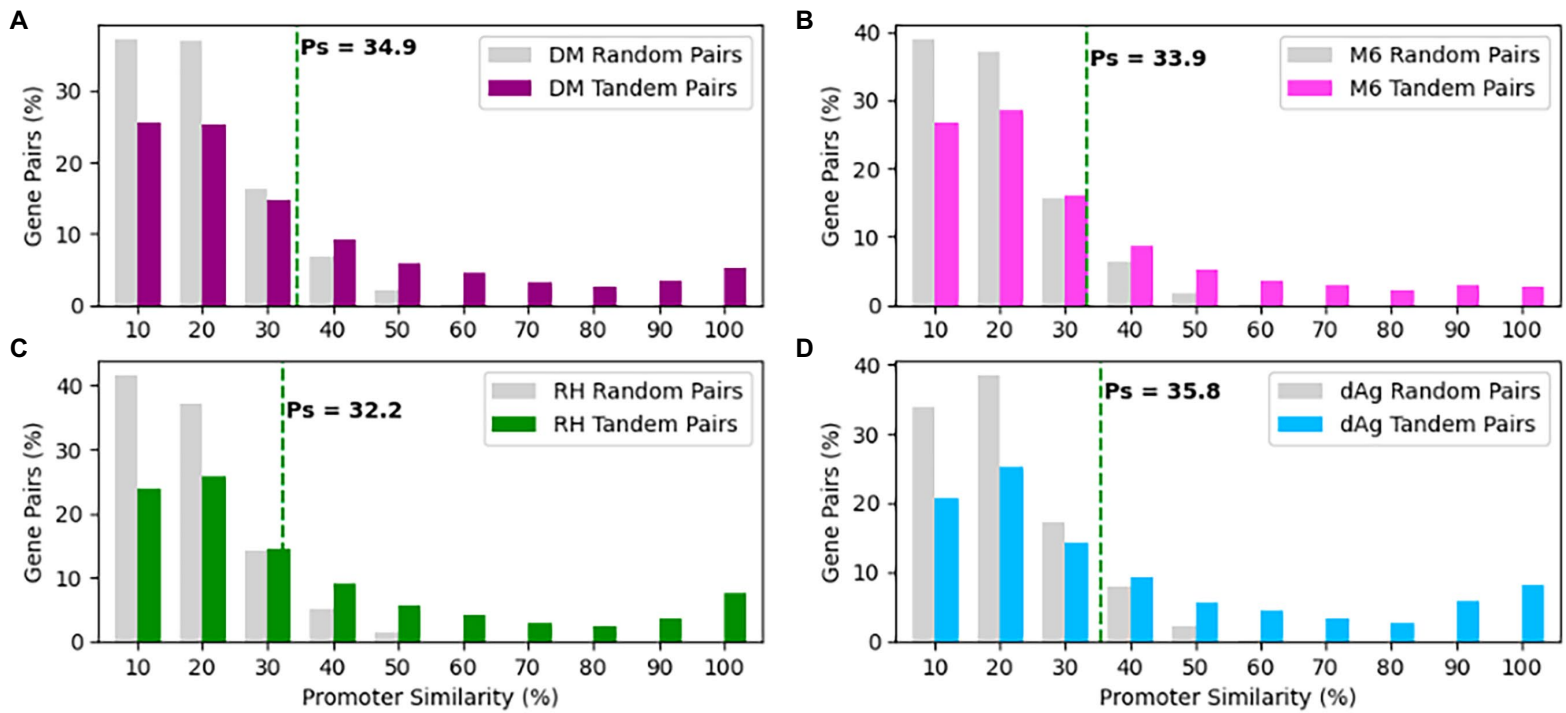
Distributions of promoter similarities (Ps) between TDG pairs in **(A)** DM, **(B)** M6, **(C)** RH, and **(D)** dAg. The dashed green line indicates 95% quantile of Ps distribution of random gene pairs. Promoter pairs with Ps < 95% quantile indicates that the promoters of the gene pairs shown to diverged, while Ps ≥ 95% quantile indicates that the promoters of the gene pair shown to conserved in respective potato genomes.

As shown above, the TDG pairs exhibited significant transcriptional divergences that prompted us to undertake a systematic investigation of variation present in their promoters. In order to do that we retrieved non-overlapping 1 Kb sequence upstream of the transcription start site for each gene of a TDG pair as a putative promoter sequence and measured promoter similarity (Ps). We measured Ps for the same number of randomly selected non-TE gene pairs of respective potato genomes to represent the background level of Ps that is expected to be observed by chance. We found a similar distribution in Ps of tandemly duplicated gene pairs across four potato genomes (Figure 7). On average, Ps for randomly selected gene pairs was 0.159, 0.152, 0.146, and 0.139%, while Ps for TDG pairs was 0.34, 0.29, 0.25, and 0.31% in dAg, DM, M6, and RH, respectively (Supplementary Table S10). As mentioned above, 95% quantile in the Ps of randomly selected gene pairs was used to classify the promoter sequences of TDG pairs as either conserved or diverged. Based on this criterion, 66.06, 70.83, 75.61, and 66.59% of TDG pairs showed diverged promoters in dAg, DM, M6, and RH, respectively (Supplementary Table S10).

### Correlation of Expression, Promoter and Protein Divergence in TDGs

**Figure 8:**
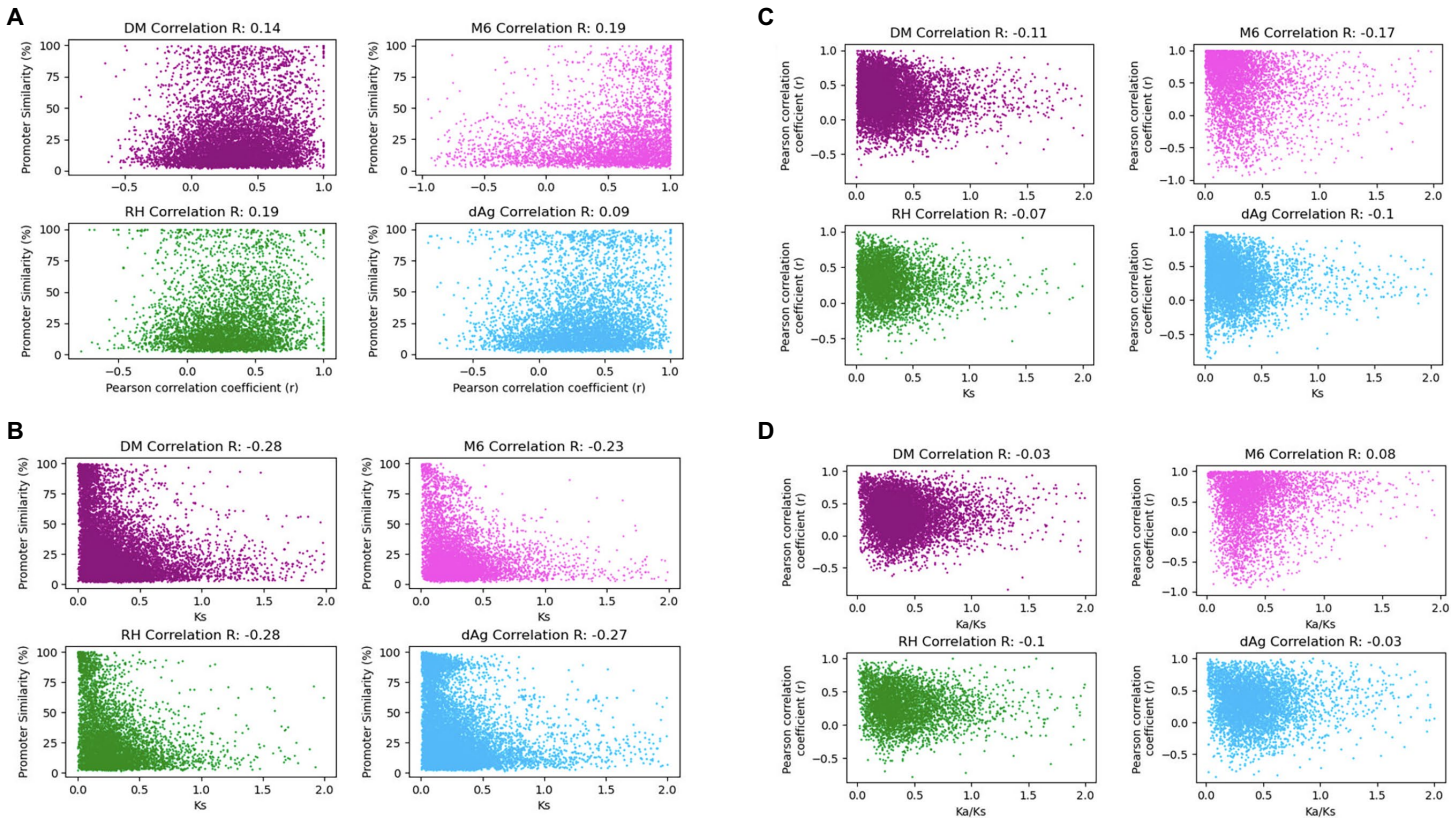
**(A)** Promoter associated with gene expression correlation (*r*). Gene expression correlation (Pearson *r*) on *x*-axis was plotted against promoter similarity within TDGs in DM, M6, RH, and dAg. **(B)** Promoter similarity coupled to protein divergence correlations. Protein divergence time *K*_s_ on *x*-axis plotted against promoter similarity within TDGs in DM, M6, RH, and dAg. **(C)** Protein divergence time *K*_s_ uncoupled to gene expression (Pearson *r*). Gene expression correlation (Pearson *r*) on *y*-axis was plotted against protein divergence time *K*_s_ within TDGs in DM, M6, RH, and dAg. **(D)** *K*_a_/*K*_s_ uncoupled to gene expression (Pearson *r*). Gene expression correlation (Pearson *r*) on *y*-axis was plotted against *K*_a_/*K*_s_ within TDGs in DM, M6, RH, and dAg.

As shown above, expression, age of TDG pairs measured as *K*_s_, and their associated promoters exhibited significant similarities as well as divergences in TDGs. It would be interesting to know the correlations among them. Therefore, to test whether the divergence of promoter similarities correlates with the expression divergence, we computed Pearson correlation coefficients (*r*) between the expressed TDG pairs against their promoter similarities. The correlation coefficients were low but significantly positive and ranged for the four genomes from 0.09 (value of *p* = 6.73 × 10^−10^) for dAg to 0.19 (value of *p* = 2.54 × 10^−39^) for RH (Figure 8A). Further, to test whether promoter divergence correlates with coding sequence divergence measured as *K*_s_, we computed Pearson correlation coefficients between *K*_s_ of TDG pairs and their respective promoter similarities. The correlation coefficients were low but significantly negative and ranged from −0.28 (value of *p* = 1.93 × 10^−198^) for DM to −0.23 (value of *p* = 7.47 × 10^−78^) for M6 (Figure 8B). However, the distribution of promoter divergence across *K*_s_ (Figure 8B) suggests a continuous expression divergence has occurred over the evolutionary time (*K*_s_) between TDG pairs. To verify this, we computed Pearson correlation coefficients between *K*_s_ of TDG pairs and their expression. Similarly, a continuous expression divergence over the time between TDG pairs occurred with a significantly negative correlation ranging from −0.17 (value of *p* = 4.97 × 10^−30^) for M6 to −0.07 (value of *p* = 6.72 × 10^−07^) for RH (Figure 8C). Further, we computed Pearson correlation coefficients between *K*_a_/*K*_s_ ratios of TDG pairs and their expression to reveal the type of selection that caused the divergence in expression between the duplicated genes of a TDG pair. The results indicated that the divergence in gene expression between duplicated genes across TDG pairs was due to purifying selection (Figure 8D). Only for M6 a positive correlation of 0.08 (value of *p* = 5.5 × 10^−8^) was observed.

### Core, Shared, and Private TDG Clusters

**Figure 9:**
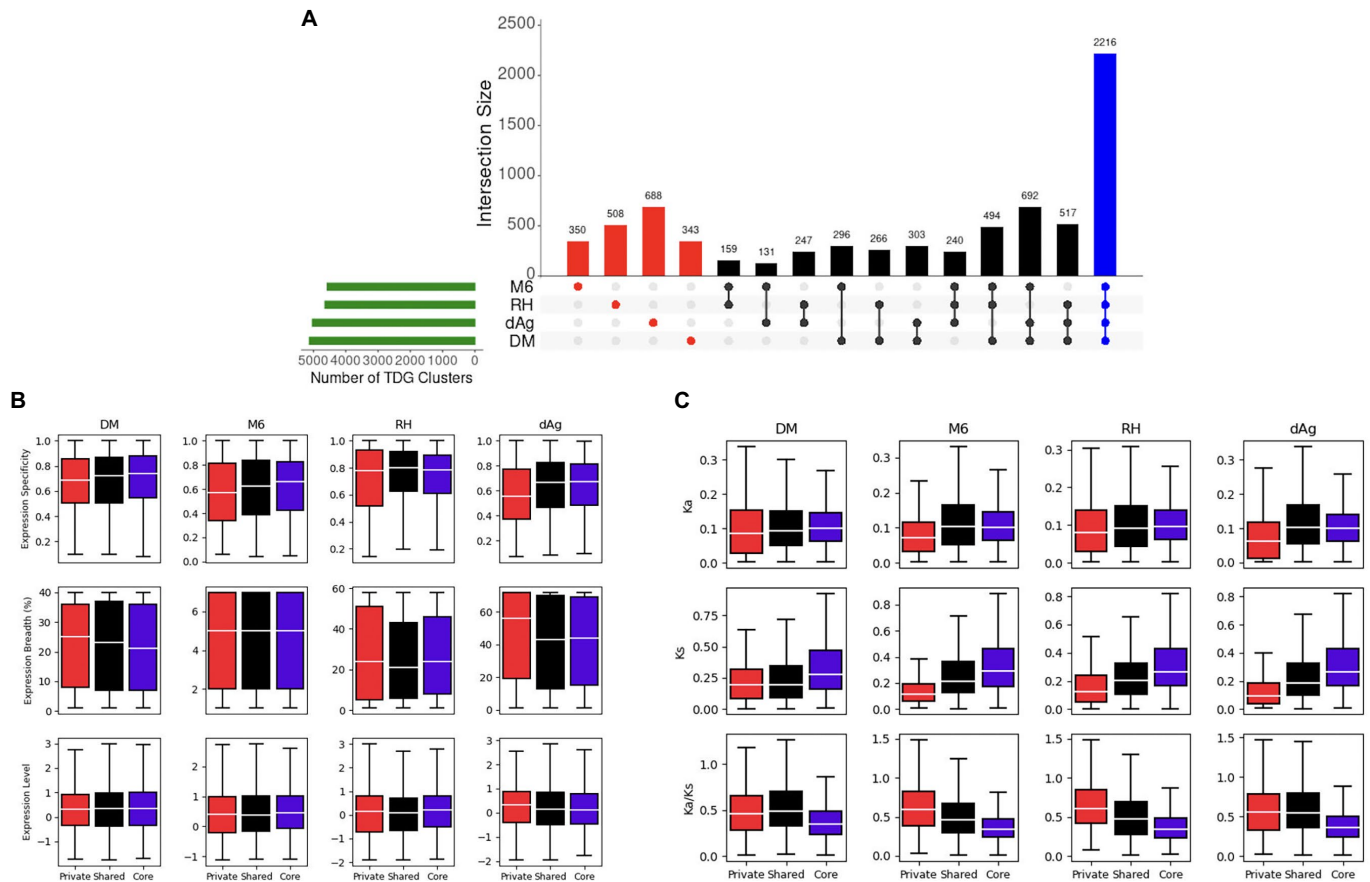
**(A)** Distribution of Private (Red), Shared (Black), and Core (Blue). Tandemly duplicated clusters across potato genomes *via* orthogroups. **(B)** Expression patterns of private, shared, and core TDG clusters. **(C)** Evolutionary patterns of private, shared, and core TDG clusters.

A total of 7,450 TDG clusters were identified across four potato genomes. To determine if TDG clusters were shared across the four potato genomes, we used the orthology information that linked the non-TE gene models of all four potato genomes and categorized them into core, shared, and private (or lineage-specific). Based on this categorization, on average, 25.02, 29.94, and 45.03% of all TDG clusters were private, core, and shared clusters, respectively, across the four potato genotypes (Figure 9A; Table 3). In addition, the non-cultivated potato genotype DM contained the highest proportion of shared clusters (51.96%), while the cultivated potato genotype dAg contained the lowest proportion of shared clusters (40.57%). Conversely, the cultivated genotype dAg contained the highest proportion of private clusters (32.92%), while the non-cultivated potato genotype DM contained the lowest proportion of private clusters (18.37%; Table 3). An average of 52.04% of Pfam protein domains enriched in all TDG clusters was present in private clusters. In addition, the private clusters of the cultivated potato genotype dAg showed with the highest proportion of enriched Pfam protein domains (about 70%), while the private clusters of the non-cultivated potato genotype RH revealed the lowest proportion of enriched Pfam protein domains (about 41%). Furthermore, an average of 62.24% of Pfam protein domains which were enriched within the private clusters were present in positively selected TDGs (*K*_a_/*K*_s_ > 1). In addition, the cultivated potato genotype dAg contained a high proportion of Pfam protein domains (about 66%) which were enriched within the private clusters and that were present in positively selected TDGs (*K*_a_/*K*_s_ > 1). In contrast, the wild potato genotype contained the lowest proportion of Pfam protein domains (about 59%) which were enriched within the private clusters and that were present in positively selected TDGs (*K*_a_/*K*_s_ > 1; Supplementary Table S11). In general, private clusters showed low-expression specificities but higher expression breadth compared to shared and core clusters. The private clusters were shown to have lower *K*_a_ and *K*_s_ values but higher *K*_a_/*K*_s_ values compared to shared and core clusters (Figure 9C). Further, we observed that the majority of private clusters localized toward the centromere, while the majority of core and shared clusters tended to localize toward the ends of chromosome-arms (Supplementary Figure S4).

**Table 3:** Number of TDG shared across potato genotypes.

Genotype	# TDG clusters	# Private	Private (%)	# Core	Core (%)	# Shared	Shared (%)
dAg	2,090	688	32.92	554	26.51	848	40.57
DM	1,867	343	18.37	554	29.67	970	51.96
M6	1,661	350	21.07	554	33.35	757	45.57
RH	1832	508	27.73	554	30.24	770	42.03

## Discussion

### Genome-Wide Identification of TDGs in Potatoes

Tandem duplications are widespread in plant genomes and contribute significantly to the evolution of genomes. By using the available well-annotated multiple *de novo* genome assemblies of potatoes, we observed that the TDGs in potato were dispersed throughout the genome with similar distribution across four potato genomes. TDGs accounted for about 18% of all non-TE genes in potatoes. This number is considerably higher than in rice (about 15.1%; Jiang et al., 2013), maize (average of 10.3%; Kono et al., 2018), and pear (about 11.1%; Qiao et al., 2018). The differences in proportions of TDGs among potatoes, rice, maize, and pear might be generated by species-specific gene duplications as observed across 141 plant species (Qiao et al., 2019). The distribution and chromosomal localization of TDGs of potato observed in our study are similar to the TDGs of rice (Jiang et al., 2013) and maize (Kono et al., 2018). Further, our results indicate that there is variation among potato genotypes for the content of TDGs in the genome (Table 2). The higher number of TDGs in dAg compared to the other three genotypes is likely due to the availability of a more accurate and larger genome assembly. For example, more than 20% of annotated non-TE genes of M6 were present on the unknown chromosome, and hence these genes were excluded from the analysis which might in turn lead to the identification of the lowest number of TDGs among the four potato genomes. However, further studies with larger number of genotypes are required in order to link the observed variation in the content of TDGs in the genome to the history of the examined genetic material.

### Differential Enrichment of Functional Specificities of TDGs

Gene duplication is a mechanism that creates functional innovation and novelty in the genome. Here, we explored the relationships between TDGs and functional specificities across cultivated and wild genotypes of potatoes. Protein domain enrichment revealed enrichment of several important protein domains related to genes involved in disease resistance [NB-ARC (PF00931) and leucine-rich repeat (PF00560; Jupe et al., 2012; Prakash et al., 2020); pathogenesis-related proteins (PF00407; Lakhssassi et al., 2020)], stress-responsive [UDP-glucosyl transferase (PF00201; Rehman et al., 2018), glutathione S-transferase (PF02798; Islam et al., 2018)], auxin-responsive protein (PF02519; Jain et al., 2006), and various biosynthetic pathways [IPP transferase (PF01715; Lindner et al., 2014)] in TDGs across four potato genomes. Our results highlighted that the potato inhibitor 1 family protein domain (PF00280) containing genes were enriched only in the wild potato genotype (i.e., M6) and were shown to be under positive selection, i.e., *K*_a_/*K*_s_ > 1 (Figure 4A). The potato inhibitor 1 family protein domain containing genes are naturally occurring plant serine proteinase inhibitors. They act as both endogenous and defense-related plant regulators in potato under wounding and nematode infection (Turrà et al., 2009). In line with protein domain enrichment, GO enrichment also revealed that these TDGs were involved in biological processes related to defense responses against various pathogens, such as bacteria, fungi, and virus, stress responses against various abiotic stress conditions (such as light, auxin, cadmium, heat, and UV), and biosynthetic pathways (lignin, saponin, phenylpropanoid, di-terpenoid, flavonoid, glucosinolate, and indole). Moreover, both protein domains and GO processes were differentially enriched across four potato genomes and TDGs encoding these functional specificities were under positive selection, i.e., *K*_a_/*K*_s_ > 1 (Figures 4A,B). These results suggested that the bias in functional specificities coupled with positive selection might play an important role in the retention of TDGs in potatoes (Shiu et al., 2006; Ren et al., 2014). This finding is consistent with a previous study that found that the retention of TDGs favors genes involved in certain important functions to maintain the fitness of the organism (Blanc and Wolfe, 2004; Edger and Pires, 2009).

### Rapid Sequence, Expression, and Regulatory Divergences Among TDG Pairs

Potato underwent at least two rounds of genome duplication, 185 and 67 million years ago (Potato Genome Sequencing Consortium et al., 2011), and retained 6,078, 5,817, 4,748, and 5,018 TDGs for dAg, DM, M6, and RH, respectively. Our study highlighted a number of striking patterns in sequence, expression, and regulatory divergences between gene copies of TDG pairs across four potato genomes (Figures 3, 5–8). Based on these patterns, we propose and distinguish multiple models such as sub-functionalization (Force et al., 1999), genetic redundancy (Panchy et al., 2016), and neo-functionalization (Ohno, 1970) that may contribute to the retention of TDGs in potato genomes.

#### Sub-Functionalization

In general, our results indicate that the TDGs were expressed in all potato genotypes in a lower number of samples but with higher expression specificities than non-TDGs in all potato genotypes (Figures 5A,B) and this in turn indicates that the duplicated genes functions in specific tissues. In addition, we found that an average of 74.43% of expressed TDG pairs showed divergence in expression (Supplementary Table S12), regardless of the age of duplication, across four potato genotypes. As we already excluded annotated partial or pseudogenes from the dataset, the divergence in expression may not be due to pseudogenization. The expression divergence is consistent with expression divergence between duplicated gene copies of TDG pairs of Arabidopsis thaliana (Haberer et al., 2004), *Glycine* max (Roulin et al., 2013), and the D-genome of *Gossypium raimondii* (Renny-Byfield et al., 2014). We also found that an average of 92.3% of expressed TDG pairs which showed divergence in expression have *K*_a_/*K*_s_ < 1.0 (Supplementary Table S12), indicative of a purifying selective pressure at the nucleotide level across four potato genotypes. In addition, either substantially a weak (for M6) or negative correlations (for dAg, DM, and RH) were observed between expression divergence and *K*_a_/*K*_s_ ratios (Figure 8D), suggesting that sub-functionalization of duplicated genes across tissues has been occurred to retain the duplicated gene copies. These results are consistent with the retention of duplicated genes through sub-functionalization in *Glycine max* (Roulin et al., 2013). Furthermore, the vast majority of the above retained TDG pairs (an average of 84.33%) had identical annotation in terms of protein domains between gene copies of respective TDG pairs (Supplementary Table S12). These results together indicated that the retention of duplicated genes occurred through sub-functionalization, where the partitioning of an ancestral gene into daughter genes across tissues implies that both daughter genes must remain functionally (Force et al., 1999; Lynch and Force, 2000). The results also demonstrated that the sub-functionalization might have been established after polyploidization in potato, and it was maintained over time (Figure 8C), as observed in *Glycine max* (Roulin et al., 2013) and cotton (Chaudhary et al., 2009).

The divergence in expression and there with sub-functionalization could be due to divergence in promoter sequences of the respective duplicated gene copies of TDG pairs (Katju and Lynch, 2003). In line with this explanation, we observed a divergence in promoter sequences of an average of 50.95% of expressed TDG pairs (Supplementary Table S12). However, only weak correlations between divergence of promoter sequences and expression levels were observed across four potato genotypes (Figure 8A). These results are consistent with a previous study in *Arabidopsis thaliana* (Haberer et al., 2004) and suggested that even small changes in the promoter sequences could be sufficient for sub- or neo-functionalization. Our results also indicated that the expression of TDGs might be regulated by trans-acting factors (Yvert et al., 2003).

#### Genetic Redundancy

Despite the overall pattern of expression divergence between duplicated gene copies of TDG pairs, for 25.6% of the expressed TDG pairs, a strong similarity in the expression profiles was observed. Of these TDG pairs, the vast majority (an average of 87.13%) was under purifying selection across four potato genotypes (Supplementary Table S12). Furthermore, with an average of 86.5% of the above retained expressed TDG pairs had an identical annotation in terms of protein domains between duplicated genes of TDG pairs (Supplementary Table S12). These results together suggested that the duplicated genes of similarly expressed TDG pairs might have been retained through selection for genetic redundancy that may be beneficial in a way that is similar to a fail-safe in engineered systems (Hanada et al., 2009; Zhang, 2012; Panchy et al., 2016). Alternatively, these TDG pairs might have been retained simply because there has been insufficient time for one copy to be removed or mutated or because they are evolving close to neutrally (Panchy et al., 2016).

#### Neo-Functionalization

Protein domain analysis performed on TDGs showed that an average of 77.56% of expressed TDG pairs contained identical protein domains between duplicated gene copies of TDG pairs across four potato genotypes. We observed that about an average of ~1% of expressed TDG pairs showed a different protein domain composition with *K*_a_/*K*_s_ > 1.0 between duplicated gene copies of TDG pairs across four potato genotypes (Supplementary Table S12). These results suggested that this ~1% of TDG pairs were retained through neo-functionalization, where both duplicate gene copies were retained because of a gain of novel functions that contributes to better fitness post duplication (Ohno, 1970). This observation is consistent with the retention of a small fraction (4%) of duplicated gene pairs through neo-functionalization in *Glycine max* (Roulin et al., 2013). Furthermore, our results also highlighted that an average of 75.91% of the retained TDG pairs showed divergence in expression (Supplementary Table S12). Overall, these results indicated that the divergence in expression, different protein functions, and positive selective pressure combinedly accounted for the neo-functionalization of those average of ~1% of TDG pairs across potato genotypes. Furthermore, we found a total of 27 enriched protein domains were present in the neo-functionalized TDG pairs across four potato genotypes and these enriched protein domains were mainly involved in important biological processes such as disease resistance (NB-ARC: PF00931, leucine-rich repeat: PF13855 and PF00560, and Rx N-terminal domain: PF18052; Jupe et al., 2012; Prakash et al., 2020); self-incompatibility (S-locus glycoprotein domain: PF00954; Xing et al., 2013); seedling development, senescence and pathogen resistance (F-box domain: PF00646; Xu et al., 2009; Supplementary Table S13).

### Private TDG Clusters Across Four Potato Genotypes

Based on the orthology information, we found a significant proportion (an average of 25.02% of all TDG clusters) of private or lineage-specific TDG clusters across four potato genotypes (Figure 9A; Table 3). The majority of them localized in pericentromeric regions which was not observed for core and shared clusters (Supplementary Figure S4). The reason for this observation might be the same that is responsible for an over-representation of presence absence variation (PAV) genes in *Arabidopsis thaliana* (Tan et al., 2012) in pericentromeric regions. The low extent of recombination in those regions of the genome might prevent the spread of present TDGs in a population, and thus the TDGs remains private. Our results highlighted that the tandem duplication generates a significantly varying proportion of private clusters across four potato genomes (Table 3). In addition, we found that the cultivated genotype dAg contained a high proportion of enriched Pfam protein domains which are present in positively selected TDGs of private clusters compared to non-cultivated as well wild potato genotypes (Supplementary Table S11). This observation might be due to breeder’s selection to combine positive alleles for many traits. These results may also indicate that the tandem duplication generates lineage-specific TDGs with functional bias between evolutionarily closed species, such as the four potato genotypes, which is similar to that of generation of lineage-specific TDGs with functional bias between evolutionarily distant plant species (Hanada et al., 2008).

In general, our results highlight that the private TDG clusters showed a lower expression specificity and higher expression breadth compare to the shared and core clusters (Figure 9B). Our observation indicates that the private clusters were involved in tissue-specific functional specificities. This result is in contrast to results of legume species (Xu et al., 2018) where private TDGs showed higher expression specificity and lower expression breadth. The reason for that remains elusive. Furthermore, an average of 30.99% of private TDG pairs showed divergence in expression and have *K*_a_/*K*_s_ < 1.0 (Supplementary Table S14) indicating purifying selection at the nucleotide level which in turn suggests that sub-functionalization of duplicated genes across tissues has been occurred to retain the duplicated genes across four potato genotypes (Force et al., 1999; Lynch and Force, 2000). In addition, a vast majority of these retained private TDG pairs (an average of 84.29%) had an identical annotation in terms of protein domains between duplicated gene copies of respective TDG pairs (Supplementary Table S14). These results together reinforced that the retention of a majority of TDGs of private clusters were occurred through sub-functionalization.

In addition, an average of 19.05% of private TDG pairs showed similarity in expression profiles, of which a majority of them (an average of 61.6%) are under purifying selective pressure (i.e., *K*_a_/*K*_s_ < 1.0), and a vast majority of them (an average of 87.62%) contained identical annotation in terms of protein domains between duplicated genes of respective private TDG pairs, across four potato genotypes (Supplementary Table S14). These results indicated that these private TDG pairs might have been retained through selection for genetic redundancy that may be beneficial in a way that is similar to fail-safe in an engineered system (Hanada et al., 2009; Zhang, 2012; Panchy et al., 2016). Alternatively, these private TDG pairs might have been retained simply because there has been insufficient time for one copy to be removed, because they are evolving relatively neutrally (Panchy et al., 2016).

We also found that an average of 0.97% only of private TDG pairs have different annotation in terms of protein domain composition with *K*_a_/*K*_s_ > 1.0 between gene copies of respective TDG pairs across four potato genotypes (Supplementary Table S14). These results indicate that these 0.97% of private TDG pairs might have been retained through neo-functionalization (Ohno, 1970). In addition, a total of eight enriched Pfam protein domains are present in these retained 0.96% of private TDG pairs in all potato genotypes and these enriched protein domains were mainly involved in important biological processes such as disease resistance (NB-ARC domain: PF00931; Leucine rich repeat: PF13855; Jupe et al., 2012; Prakash et al., 2020) and self-incompatibility (S-locus glycoprotein domain: PF00954; Xing et al., 2013; Supplementary Table S15).

### Lineage-Specific Expansion of Gene Families and Species Divergences

Our results indicated that the tandem duplication contributed to lineage-specific expansion of several gene families across potato genotypes. For example, NBS-LRR, Cytochrome P450, UDP-glucosyl transferase, and 2OG-Fe (II) oxygenase gene families were differentially expanded by tandem duplication across potato genotypes (Figure 4A). Furthermore, the GO enrichment revealed a functional bias of TDGs across the four potato genotypes (Figure 4B). This is supported by recent studies of specific gene families in potato (Herath and Verchot, 2020; Liu et al., 2020; Yang et al., 2020; Xuanyuan et al., 2022) and provided an important source for genetic diversity in plants for adaptive evolution against various environmental stimuli. These results are similar to a previous study conducted on two maize genotypes (such as B73 and PH207) where more than 49% of B73’s and 40% of PH207’s TDGs were lineage-specific (Kono et al., 2018). Furthermore, the importance of lineage-specific expansion of TDGs was also studied in *A. thaliana* against various abiotic stress stimuli and found a strong correlation between tandem duplication and abiotic stress conditions (Hanada et al., 2008). Thus, the lineage-specific expansion of gene families by tandem duplication coupled with functional bias might significantly contribute to potato’s genotypic diversity. However, to understand their effect on phenotypic characters requires further research.

## Conclusion

By investigating the divergence in sequence, functional and transcriptional features of TDGs across four diploid potato genomes, we found that after at least two rounds of genome duplication, a large proportion of TDGs were retained through sub-functionalization. Sub-functionalization, by keeping both copies of the same gene, may pave an intermediate step to neo-functionalization for some genes, which is supported by a very small fraction of neo-functionalized duplicated TDGs in potatoes. In addition, TDGs contributed to lineage-specific expansion of several gene families for adaptive changes. These results show that evolution of functions and fates of genes after tandem duplication is a complex process which drives the evolution of gene duplication in association with expression, as well as the duplicated and/or retention of genes with specific functions. In addition, we found variation within TDGs among cultivated, non-cultivated and wild potato genotypes in terms of bias in functional specificities, proportion of lineage-specific clusters, diverged expression and promoter similarities.

## Data Availability Statement

Publicly available datasets were analyzed in this study. This data can be found at: https://www.figshare.com/s/7dee6e184ab4cc666976.

## Author Contributions

VSB conceived, designed, performed the experiments and data analysis, and wrote the manuscript. BS contributed to data analysis and manuscript writing. All authors contributed to the article and approved the submitted version.

## Conflict of Interest

The authors declare that the research was conducted in the absence of any commercial or financial relationships that could be construed as a potential conflict of interest.

## Publisher’s Note

All claims expressed in this article are solely those of the authors and do not necessarily represent those of their affiliated organizations, or those of the publisher, the editors and the reviewers. Any product that may be evaluated in this article, or claim that may be made by its manufacturer, is not guaranteed or endorsed by the publisher.

## Footnotes

^1^ https://github.com/groupschoof/AHRD

^2^ https://www.r-project.org

^3^ https://www.ncbi.nlm.nih.gov/sra

^4^ https://www.python.org

^5^ https://github.com/groupschoof/AHRD

## Supplementary Material

Supplementary Figure S1 Distribution of number of exons within TDGs.

Supplementary Figure S2 Distribution of number of Pfam domains in TDGs.

Supplementary Figure S3 Enrichment of Pfam protein domains in all TDGs.

Supplementary Figure S4 Distribution of core, shared, and private tandemly duplicated gene clusters across the genomes of **(A)** DM, **(B)** M6, **(C)** RH, and **(D)** dAg.

Supplementary Figure S5 Distribution of density of tandemly duplicated genes per 1.5Mb across each potato genome. Square boxes between rows do not correspond to sequence alignment
