# Supplementary figures and images for: Genetic Divergence of Lineage-Specific Tandemly Duplicated Gene Clusters in Four Diploid Potato Genotypes
Source: Front Plant Sci. 2022 May 11;13:875202. doi: 10.3389/fpls.2022.875202 (PMC9131075; doi:10.3389/fpls.2022.875202)

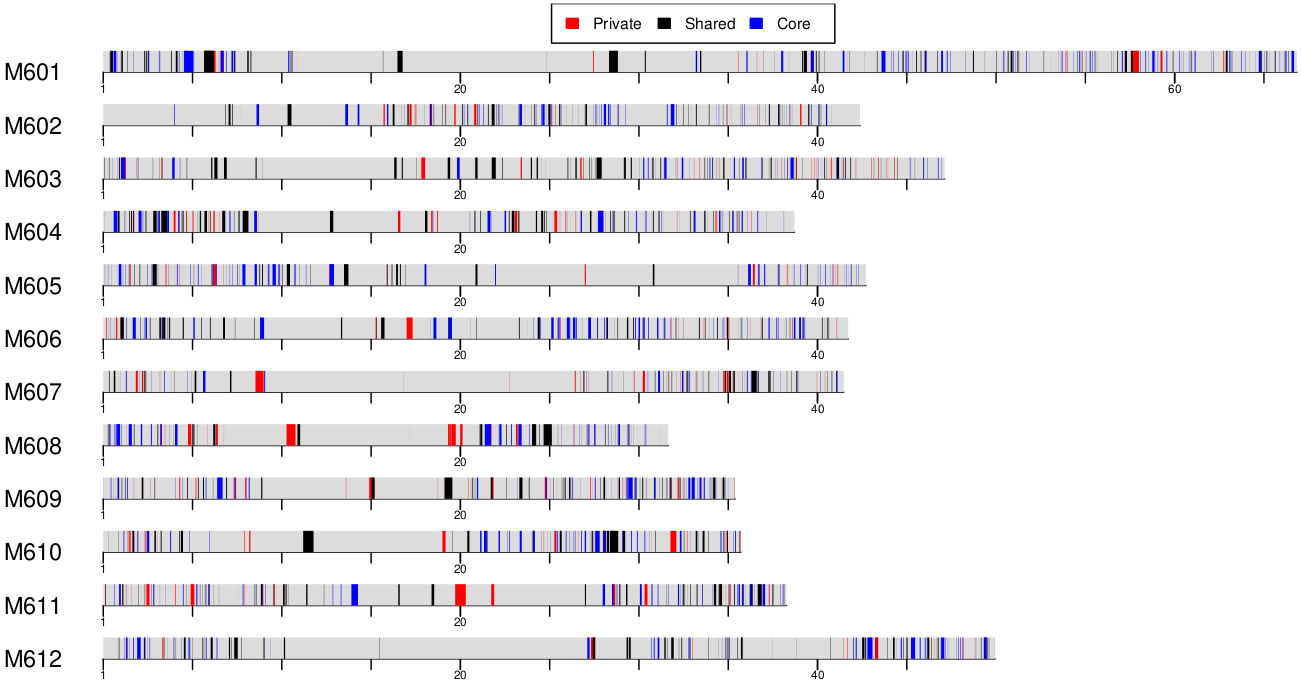

Supplement: Supplementary Figure S1 — Distribution of number of exons within TDGs. [file Data_Sheet_1.ZIP › Supplementary_Material/Figures/Figure S4B.tif]

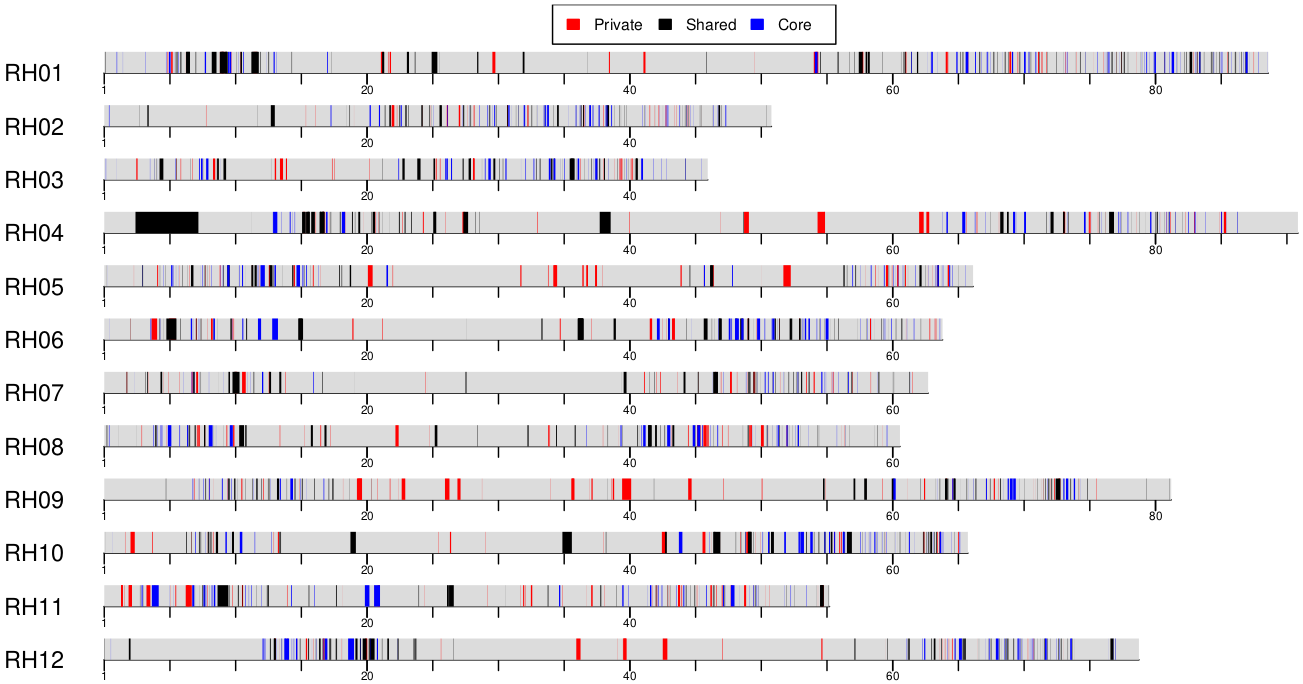

Supplement: Supplementary Figure S1 — Distribution of number of exons within TDGs. [file Data_Sheet_1.ZIP › Supplementary_Material/Figures/Figure S4C.tif]

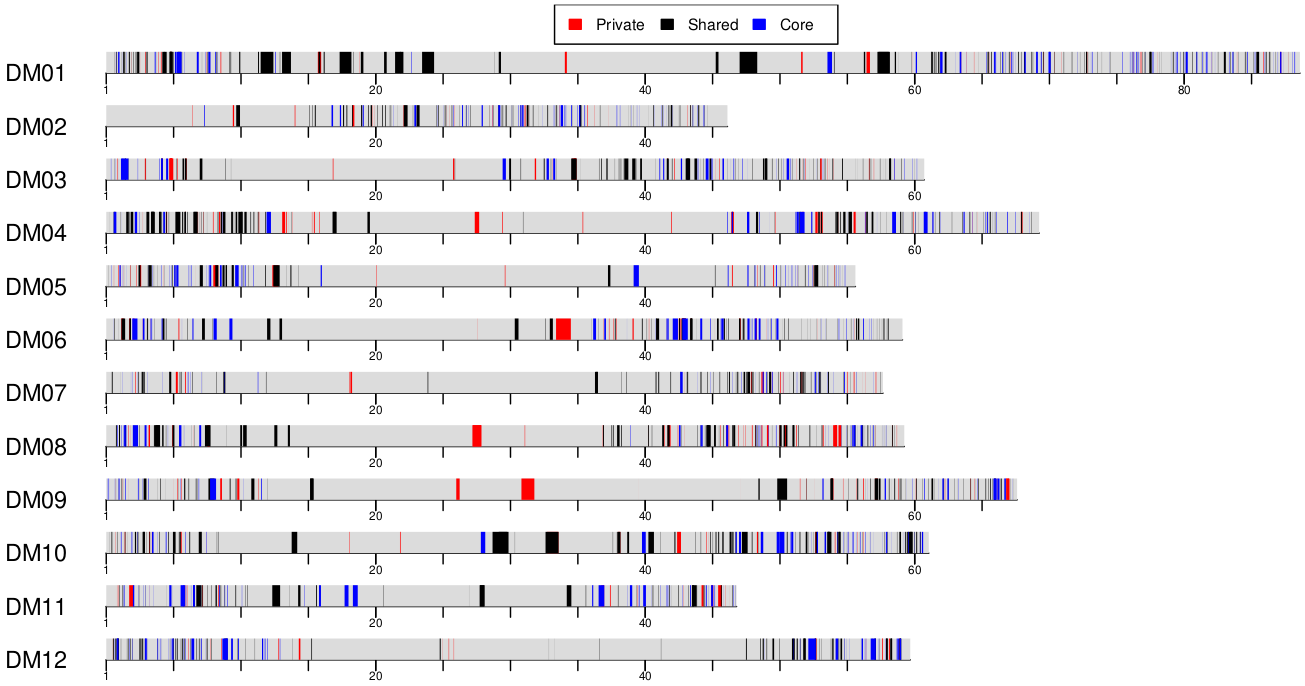

Supplement: Supplementary Figure S1 — Distribution of number of exons within TDGs. [file Data_Sheet_1.ZIP › Supplementary_Material/Figures/Figure S4A.tif]

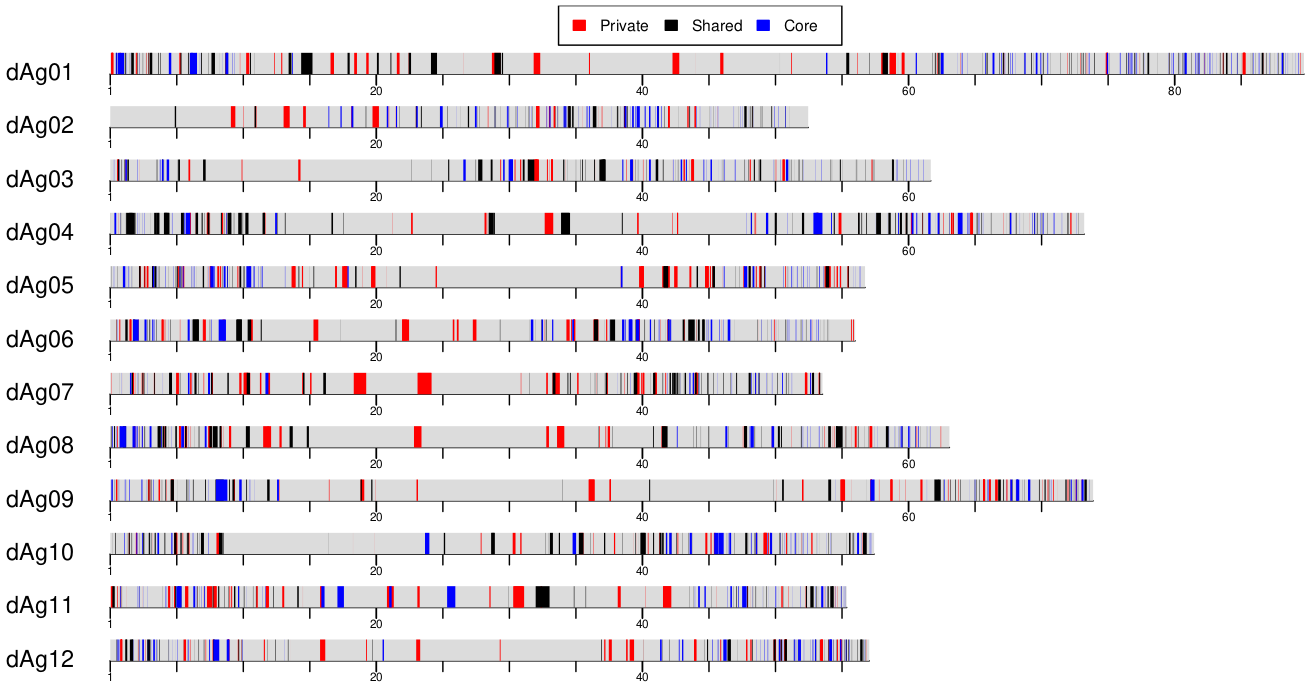

Supplement: Supplementary Figure S1 — Distribution of number of exons within TDGs. [file Data_Sheet_1.ZIP › Supplementary_Material/Figures/Figure S4D.tif]

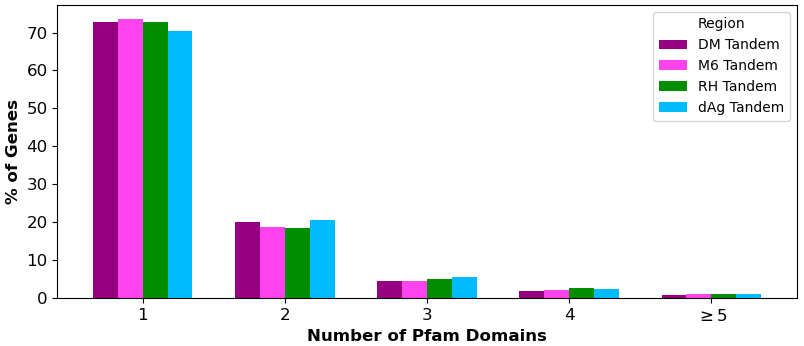

Supplement: Supplementary Figure S1 — Distribution of number of exons within TDGs. [file Data_Sheet_1.ZIP › Supplementary_Material/Figures/Figure S2.tif]

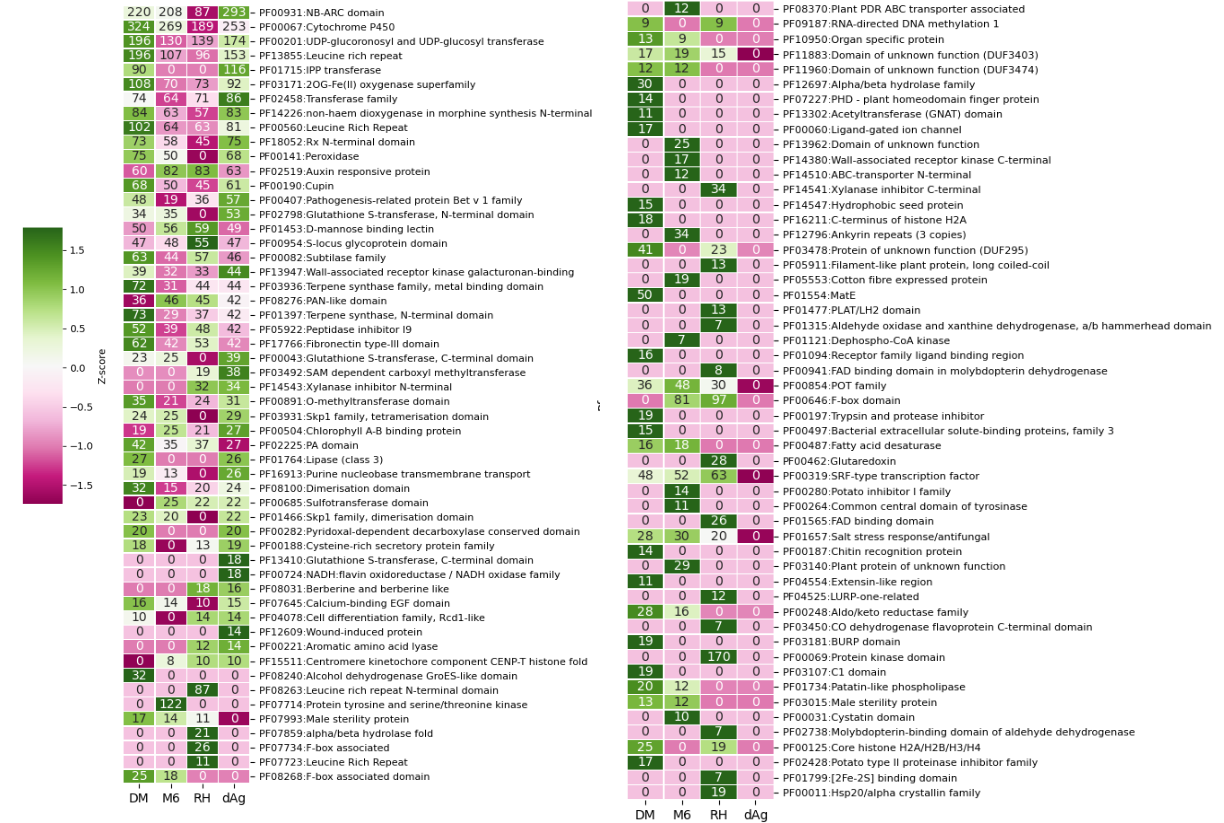

Supplement: Supplementary Figure S1 — Distribution of number of exons within TDGs. [file Data_Sheet_1.ZIP › Supplementary_Material/Figures/Figure S3.tif]

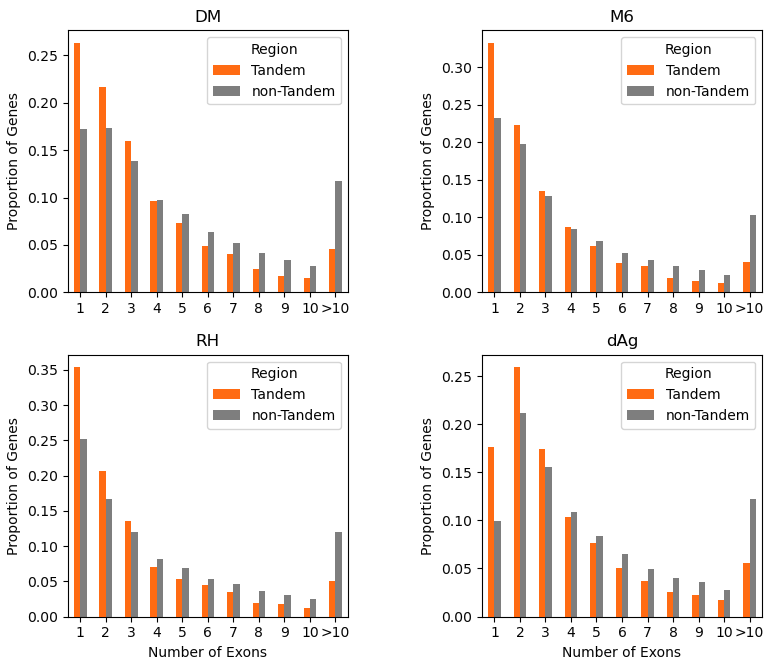

Supplement: Supplementary Figure S1 — Distribution of number of exons within TDGs. [file Data_Sheet_1.ZIP › Supplementary_Material/Figures/Figure S1.tif]
